# Immune - cell death index in hepatocellular carcinoma: a multi-omics and machine learning study for prognosis and immunotherapy prediction

**DOI:** 10.3389/fimmu.2026.1776723

**Published:** 2026-06-11

**Authors:** Yi Zhang, Haiyu Zhao, Yunpeng Zhai, Chongyang Wang, Zhenya Wang, Ruopeng Liang

**Affiliations:** 1 The First Clinical Medical College, Zhengzhou University, Zhengzhou, Henan, China; 2 Department of Hepatobiliary and Pancreatic Surgery, The First Affiliated Hospital of Zhengzhou University, Zhengzhou, Henan, China

**Keywords:** hepatocellular carcinoma, immunotherapy efficacy, machine learning, multi-omics analysis, prognosis, programmed cell death

## Abstract

The heterogeneity of hepatocellular carcinoma (HCC) and individual disparities in immunotherapy response necessitate the urgent development of accurate evaluation tools. Programmed cell death (PCD) is implicated in the occurrence and development of HCC. Moreover, immune-related genes have a crucial role in cancer progression and patient prognosis. This study employed 10 clustering algorithms to conduct high-resolution molecular subtyping based on PCD-related genes, immune-related genes, microRNA, long non-coding RNA, and methylation data. Subsequently, we developed hepatocellular carcinoma consensus immune-cell death index (HICDI) by employing subtype-specific genes and merging 10 commonly used machine learning algorithms into 101 unique combination frameworks. Our HICDI score exhibited enhanced predictive ability compared to previously published HCC biomarkers. Patients with a low HICDI score exhibited higher overall survival and improved responses to immunotherapy. The high HICDI group exhibited a propensity for “cold” tumors marked by immune suppression and exclusion; however, drugs such as paclitaxel may present viable therapeutic options for these patients. We verified the model gene kinesin family member 2C through *in vitro* experiments, demonstrating its role as a potential oncogene affecting HCC progression and as a promising therapeutic target. Overall, HICDI possesses the potential for extensive applications in informing personalized treatment decisions and improving outcomes for patients with HCC.

## 1 Introduction

Hepatocellular carcinoma (HCC) is the sixth most common malignancy and the third leading cause of cancer-related mortality worldwide. In 2020, approximately 906,000 new cases and 830,000 deaths were reported, of which over 75% occurred in Asia (1). Despite advancements in immunological combination therapies enhancing the objective response rate (ORR), over 50% of patients remain unresponsive, and the 5-year survival rate for advanced HCC remains below 20%. Resistance mechanisms encompass an immunosuppressive milieu, characterized by regulatory T (Treg) cell infiltration and myeloid-derived suppressor cell proliferation and loss of tumor antigens (2). Furthermore, patients with advanced HCC often have cirrhosis and impaired liver function, constraining systemic treatment options. Hyperprogression in immune checkpoint inhibitor (ICI) treatment (10%–15%) and the lack of effective biomarkers, exemplified by the weak correlation between programmed death-ligand 1 (PD-L1) expression and efficacy, pose major challenges (3). The primary challenge in managing HCC lies in its high molecular heterogeneity and individual disparities in treatment response. To overcome this challenge, it is imperative to develop highly specific biomarkers and predictive models. A multi-dimensional feature analysis technique offers potential to overcome the shortcomings of conventional single-dimensional biomarkers and advance the clinical translation of precision medicine for HCC.

Programmed cell death (PCD) is a gene-regulated mechanism of active cellular death that is essential for preserving tissue homeostasis, embryonic development, and immunological regulation (4). PCD includes numerous types, including apoptosis, ferroptosis, autophagy, pyroptosis, cuproptosis, necroptosis, parthanatos, netotic cell death, entotic cell death, alkaliptosis, lysosome-dependent cell death, oxeiptosis, neutrophil extracellular trap–related cell death, immunogenic cell death (ICD), anoikis, paraptosis, methuosis, entosis, mitochondrial permeability transition-driven necrosis, and disulfidptosis (5–7). Apoptosis, a gene-regulated type of PCD, is primarily controlled by two pathways: the mitochondrial pathway (Bcl-2/Bax regulating cytochrome c release) and the death receptor pathway (Fas/Fas-associated death domain protein activating caspase-8), which are crucial for embryonic development, immune homeostasis, and tissue remodeling. Ferroptosis, characterized by iron-dependent lipid peroxidation, is marked by the depletion of glutathione (GSH) and the inactivation of GSH peroxidase 4 (GPX4), leading to lipid peroxidation-induced membrane damage. This mechanism is regulated by System Xc^-^ (SLC7A11/SLC3A2) and lipid metabolism enzymes (ACSL4/LPCAT3) and operates independently of caspase or necroptosis pathways. Ferroptosis plays a significant role in cancer resistance, neurodegenerative diseases, and ischemia-reperfusion injury (8). ICD is a specific type of PCD characterized by the release of damage-associated molecular patterns, including calreticulin, high-mobility group box 1, and ATP, which stimulate dendritic cells and initiate antitumor T-cell immune responses. ICD enhances tumor antigen presentation and the efficacy of immune checkpoint inhibitors (9–11). Pyroptosis, mediated by inflammasome activation and Gasdermin proteins, is marked by cell membrane pore formation and the release of pro-inflammatory cytokines (interleukin [IL]-1β and IL-18), triggering a strong inflammatory response. Targeting the pyroptosis pathway (inhibiting GSDMD) has become a novel approach for treating inflammatory diseases (12, 13). Autophagy, a conserved self-renewal mechanism in eukaryotic cells, entails the lysosomal destruction of damaged intracellular constituents (including protein aggregates and impaired organelles). Its primary functions encompass regulating energy homeostasis, eliminating harmful substances, and responding to stress. Moderate activation can inhibit tumor occurrence and neurodegenerative disorders, such as Alzheimer’s disease; however, excessive activation may result in chemotherapy resistance or enhanced cancer cell survival. Targeting autophagy regulators is emerging as a promising treatment option for cancer and metabolic diseases (14–17). Furthermore, several types of PCD, including cuproptosis and disulfidptosis, have been extensively investigated in HCC; however, their roles in HCC remain unclear.

Cancer subtype classification based on PCD molecular features is essential for understanding the heterogeneity of HCC and its implications for diagnosis, prognosis, and treatment strategies. This study identified two molecular subtypes of HCC through the application of a consensus of 10 clustering algorithms on integrated transcriptomic, epigenetic, and somatic mutation data. A comparative analysis of the inter-subtype heterogeneity subsequently highlighted key events in hepatocellular carcinogenesis. Furthermore, we successfully developed an HCC multi-omics signature (HICDI) comprising 10 genes utilizing 10 machine learning algorithms. Our findings revealed the superior efficacy of this signature in forecasting survival prognosis and immunotherapy response of patients with HCC, establishing a key basis for precision subtyping based on molecular mechanisms and optimizing clinical treatment strategies for HCC. Through a series of experiments, we confirmed that kinesin family member 2C (KIF2C) enhances tumor proliferation and metastasis in HCC, identifying KIF2C as a crucial regulatory factor in HCC progression, and its targeted intervention presents potential for a novel technique for the accurate treatment of HCC.

## 2 Methods

### 2.1 Data acquisition and processing

Multi-omics data of liver hepatocellular carcinoma (LIHC), including transcriptome expression, DNA methylation, somatic mutations, and clinical data, were obtained from The Cancer Genome Atlas (TCGA) (https://portal.gdc.cancer.gov/) via the TCGA biolinks (18). After integrating transcriptome and methylation data of HCC patients, the final multi-omics analytical cohort comprised 326 HCC patients. Using the TCGA biolinks R package, cBioPortal, and the GEOquery R package (19), we acquired the transcriptome data, genomic data, and clinical data of HCC and other tumors from TCGA, the International Cancer Genome Consortium (ICGC) database, cBioPortal, and the Gene Expression Omnibus database. In total, 10 cancer datasets (TCGA-LIHC, ICGC-LIRI, GSE14520, TCGA-LUAD, TCGA-BLCA, TCGA-BRCA, TCGA-HNSC, TCGA-SARC, TCGA-SKCM, and TCGA-UCEC) with transcriptome expression and complete clinical outcomes were included. Three immunotherapy cohorts (IMvigor210 cohort, GSE100797, and GSE35640) and a cohort of transarterial chemoembolization (TACE)-treated patients (GSE104580). Additionally, we acquired one single-cell dataset (GSE166635) and one spatial transcriptomics dataset of HCC from (https://data.mendeley.com/datasets/skrx2fz79n/1). Tumor immune dysfunction and exclusion (TIDE) scores were downloaded from the publicly available TIDE database (http://tide.dfci.harvard.edu) for predicting immune checkpoint blockade responses in LIHC. Transcript expression values were normalized to transcripts per million (TPM) and log-2 transformed. Batch effects in merged datasets were corrected using the Combat function from the Surrogate Variable Analysis package (20).

### 2.2 Dentification of gene signatures associated with immunity and PCD

We obtained immune-related genes from the Immunology Database and Analysis Portal database and acquired 20 PCD modalities and their key regulatory genes from previously published literature (21).

### 2.3 Multi-omics data integration and clustering

Multi-omics datasets, including PCD-related mRNA expression, IRG mRNA expression, microRNA expression, long non-coding RNA (lncRNA) expression, and DNA methylation data, were integrated for molecular subtyping of LIHC using the MOVICS R package (22). Additionally, 10 clustering algorithms (SNF, CIMLR, ConsensusClustering, PINSPlus, iClusterBayes, NEMO, moCluster, IntNMF, LRA, and COCA) were applied, and the optimal number of clusters was selected based on clustering prediction index (CPI) metrics and differential statistics. We defined two consensus molecular subtypes to stratify HCC patients.

### 2.4 Characteristics of molecular subtyping and stability of consensus clustering

To delineate the subtype-specific molecular signatures and underlying regulatory mechanisms, we utilized the PROGENy package to assess the activity of oncogenic pathways in HCC patients (23). Hallmark gene sets were retrieved from the Molecular Signatures database (https://www.gseamsigdb.org/gsea/msigdb), and pathway enrichment scores were calculated using the gsva function for gene set variation analysis (GSVA) in the GSVA R package (24). Transcriptional regulatory networks were developed using the Regulatory Transcriptional Network R package (25), which included 23 transcription factors (TFs) connected to their activated or repressed target genes together with 68 putative regulatory factors implicated in chromatin remodeling. Subsequently, we quantified the transcript abundance of key immune checkpoint ligands and receptors across subtypes and calculated the stromal and immune scores for each sample utilizing the ESTIMATE algorithm (26). Simultaneously, tumor-infiltrating lymphocyte levels were assessed using a standardized DNA methylation analysis workflow (27). Additionally, we utilized the built-in functions of the MOVICS package to calculate the fraction of the genome (FGA) altered by copy number amplification or deletion. To evaluate the robustness of the molecular subtyping, we compared the Nearest Template Prediction (NTP) classifier (constructed using the top 200 upregulated genes in CS1 and CS2) with the Partitioning Around Medoids (PAM) algorithm with the consensus subtypes (CSs). We further confirmed the clustering results in an NTP-based validation cohort and analyzed survival differences between subtypes using Kaplan–Meier (K-M) curves.

### 2.5 Assessment of immune cell abundance

To quantify immune cell abundance in the samples, we employed the MCP-counter R package (28), which infers immune cell infiltration based on transcriptomic profiles. Using the IOBR R package (29), we calculated enrichment scores for each sample according to established gene signatures associated with immunotherapy response, immune suppression, and immune exclusion, applying a standardized method. To further characterize immunological differences between patients with high and low HICDI, we applied the ssGSEA function from the GSVA R package to score immune cell infiltration.

### 2.6 Determining drug sensitivity among different subtypes and risk groups

Drug sensitivity across molecular subtypes was evaluated using the built-in functions of the MOVICS package. Potential therapeutic agents for high-risk patients were identified using the Cancer Therapeutics Response Portal (CTRP) 2.0 database (https://portals.broadinstitute.org/ctrp) and Profiling Relative Inhibition in Mixtures (PRISM) (https://depmap.org/portal/prism/) databases. These databases estimate tumor drug sensitivity by evaluating area under the curve (AUC) values.

### 2.7 Integrating machine learning algorithms to construct optimal signature

To comprehensively explore the association between molecular typing and clinical prognosis, as well as to identify clinically applicable prognostic biomarkers, we first employed univariate Cox regression models to screen for biomarkers that are upregulated in each subtype and possess prognostic significance. Subsequently, we applied 10 distinct machine learning algorithms and evaluated 101 potential algorithm combinations. These machine learning algorithms included random survival forests, elastic net (Enet), Lasso, Ridge, stepwise Cox, CoxBoost, partial least squares regression for Cox regression (plsRcox), supervised principal components (SuperPC), gradient boosting machine (GBM), and survival support vector machine (survival-SVM). Using these algorithms, we utilized the TCGA cohort as the training cohort for prognostic model development, and all models were validated for their performance in two independent cohorts (ICGC-LIRI and GSE14520). Ten-fold cross-validation was adopted to mitigate potential overfitting during the model development process. Specifically, the training dataset was randomly divided into 10 equally sized folds. In each iteration, 9 folds were used for model training, and 1 fold was used for validation. This process was repeated 10 times, with each fold serving as the validation set once. This cross-validation process was consistently applied to all algorithm combinations to ensure fair comparison. We calculated Harrell’s concordance index (C-index) and its mean value for each model. In the selection of the final prognostic model, two main criteria were comprehensively considered: (1) a high mean C-index ranked within the top 5% among all established models; (2) model practicality, represented by the number of genes ultimately incorporated into the model. According to the above criteria, StepCox[both] combined with SuperPC was finally determined as the optimal prognostic model with the best comprehensive performance.

### 2.8 scRNA data processing and analysis

A Seurat object was constructed using the Seurat R package (30), retaining cells that met the following quality-control criteria: 200–5000 detected genes, log_10_ (genes per UMI) > 0.8, and a mitochondrial gene proportion ≤ 15%. All data were batch-corrected using the Harmony package before and after integration. After dimensionality reduction through principal component analysis, the first 30 principal components were selected for UMAP visualization. Cell clustering was conducted using the FindClusters function, and cell types were annotated based on established marker genes reported in the literature. Differentially expressed genes were identified using FindAllMarkers and FindMarkers functions and the COSG R package. Subsequently, we utilized the CytoTRACE tool to estimate cell developmental potential, where higher scores reflect greater differentiation potential and higher gene expression diversity (31). Additionally, Slingshot was employed to investigate the differentiation directions of malignant cells (32). To investigate potential transcriptional regulatory networks in malignant cells, we performed transcription factor module analysis using the decoupleR package (33).

### 2.9 Cell communication analysis

We utilized the CellChat package (34) to assess cell-cell communication between malignant cells with high HICDI scores and other components of the tumor microenvironment (TME). First, we calculated the overall communication strength and frequency among all cell types and malignant cells with high HICDI scores. Subsequently, we identified key cell types interacting with malignant cells with high HICDI scores and predicted potential receptors and ligands.

### 2.10 Spatial transcriptome data analysis

We directly utilized the processed data from previous studies. These datasets had already undergone normalization and, in some cases, included complete cell-type annotations. For instance, data from Liu’s group contained fully annotated cell labels. Subsequently, we scored each spot in the data using the GSVA algorithm based on HICDI genes.

### 2.11 HICDI‐based screening of potential therapeutic agents

We obtained drug sensitivity data from the CTRP database (https://portals.broadinstitute.org/ctrp) and the PRISM drug-repositioning dataset (https://depmap.org/portal/prism/). Both datasets provide AUC values, where a lower AUC value indicates higher cellular sensitivity to the compound. Differences in drug responses between the high HICDI group and the low HICDI group were assessed using the Wilcoxon rank-sum test, with a threshold of log2 fold change (log2FC) > 0.1. Subsequently, we filtered compounds that met the following criteria: AUC values were negatively correlated with HICDI scores (assessed by Spearman correlation test), with a correlation coefficient R < -0.3. Based on the above analysis results, we identified potential drugs specifically applicable to patients in the high HICDI group.

### 2.12 Molecular docking analysis of drugs and target protein

Based on the potential drugs identified through the transcriptome analysis, we performed molecular docking between lofarabine and the KIF2C protein using CB-Dock2 (https://cadd.labshare.cn/cb-dock2/php/index.php). This analysis validated the binding interaction between KIF2C and clofarabine, further supporting the reliability of our previous results.

### 2.13 Cell lines and cell culture

Human HCC cell lines (Huh7 and MHCC97H) were obtained from Procell. The cells were cultured in Dulbecco’s modified Eagle medium (DMEM) and minimum essential medium supplemented with 10% fetal bovine serum (FBS) at 37 °C with 5% CO2. The cell density was maintained between 5 × 105 and 2 × 106 cells/mL. Mycoplasma detection was performed, and no contamination was found.

### 2.14 Cell transfection

Small interfering RNA (siRNA) targeting KIF2C and control siRNA were designed by Shanghai Obio Technology (Group) Corp., Ltd. (Shanghai, China). For transient transfection, siRNA was transfected into Huh7 and MHCC97H cells utilizing a transfection reagent (Lipofectamine 2000) for 12 h, followed by functional assays and subsequent experiments.

### 2.15 Cell migration and invasion analysis

Cell migration and invasion were assessed utilizing Transwell chambers (pore size 8.0 μm, catalog number 3422; Corning Inc., USA). The lower chamber was filled with 600 μL of culture medium containing 20% FBS as a chemoattractant, whereas the upper chamber contained 200 μL of serum-free DMEM with suspended cells. For the invasion assay, the polycarbonate membrane of the upper chamber was pre-coated with Matrigel matrix (2 mg/mL, catalog number 356234, BD Biosciences, USA). Transfected cells were seeded into the upper chamber at 5 × 104 cells/mL and cultured for 24 h at 37 °C with 5% CO2. After incubation, cells in the lower chamber were fixed with 4% paraformaldehyde and stained with 0.01% crystal violet solution (Wuhan Servicebio Technology Co., Ltd., China). The number of cells traversing the membrane was quantified using an optical microscope (model DP74; Olympus Corporation, Japan), evaluating six randomly selected fields per sample for statistical analysis.

### 2.16 Western blot analysis

The harvested tissues were homogenized and lysed with radioimmunoprecipitation assay (RIPA) buffer (P0013B, Beyotime, China) for 20 min, followed by centrifugation at 13,500 rpm for 15 min. Cell lysates were prepared utilizing the RIPA method. Proteins were separated via sodium dodecyl sulfate–polyacrylamide gel electrophoresis (PG112, Epizyme Biotech, Shanghai, China) and transferred to polyvinylidene fluoride membranes (ISEQ00010, Millipore, USA). The KIF2C antibody was procured from Wuhan Sanying at a dilution of 1:1000, the β-actin antibody at a dilution of 1:2000, and the secondary antibody at a dilution of 1:10000.

### 2.17 Colony formation

A density of 500 cells per well was employed to inoculate the HCC cells onto six-well plates. We replaced the cell culture media every three days. After 12 days, the colonies were fixed for 15 min in 4% paraformaldehyde before staining with crystal violet. We employed ImageJ to quantify the colonies in each well, subsequent to capturing their images.

### 2.18 Wound healing

We cultured the HCC cells in a medium supplemented with 10% FBS for 24 h following their seeding in six-well plates until 90% confluence was achieved. Subsequently, serum-free media were introduced to the wells after the linear scraping of the cells along their diameter using a 1000 μL pipette tip. An inverted microscope was used to photograph the wound at 0 and 48 h post-injury, subsequently measuring the extent of healing.

### 2.19 Statistical analysis

Data processing, statistical analyses, and visualization were conducted using R software (version 4.4.0). The unpaired Student’s t-test was utilized for comparisons between groups when the variables were normally distributed. The Wilcoxon rank-sum test was utilized when variables had a non-normal distribution. The Kruskal–Wallis test was utilized for non-parametric variables in comparisons involving several groups, whereas one-way analysis of variance (ANOVA) was utilized for parametric variables. The log-rank test was utilized to evaluate survival disparities using K-M curves, with statistical significance established at p < 0.05. Adjusted p-values for multi-omics feature analysis, immune cell scoring, and drug screening were obtained using the Benjamini–Hochberg (BH) method.

## 3 Results

### 3.1 Identification of two key molecular subgroups associated with prognosis in HCC via a multiomics approach

**Figure 1:**
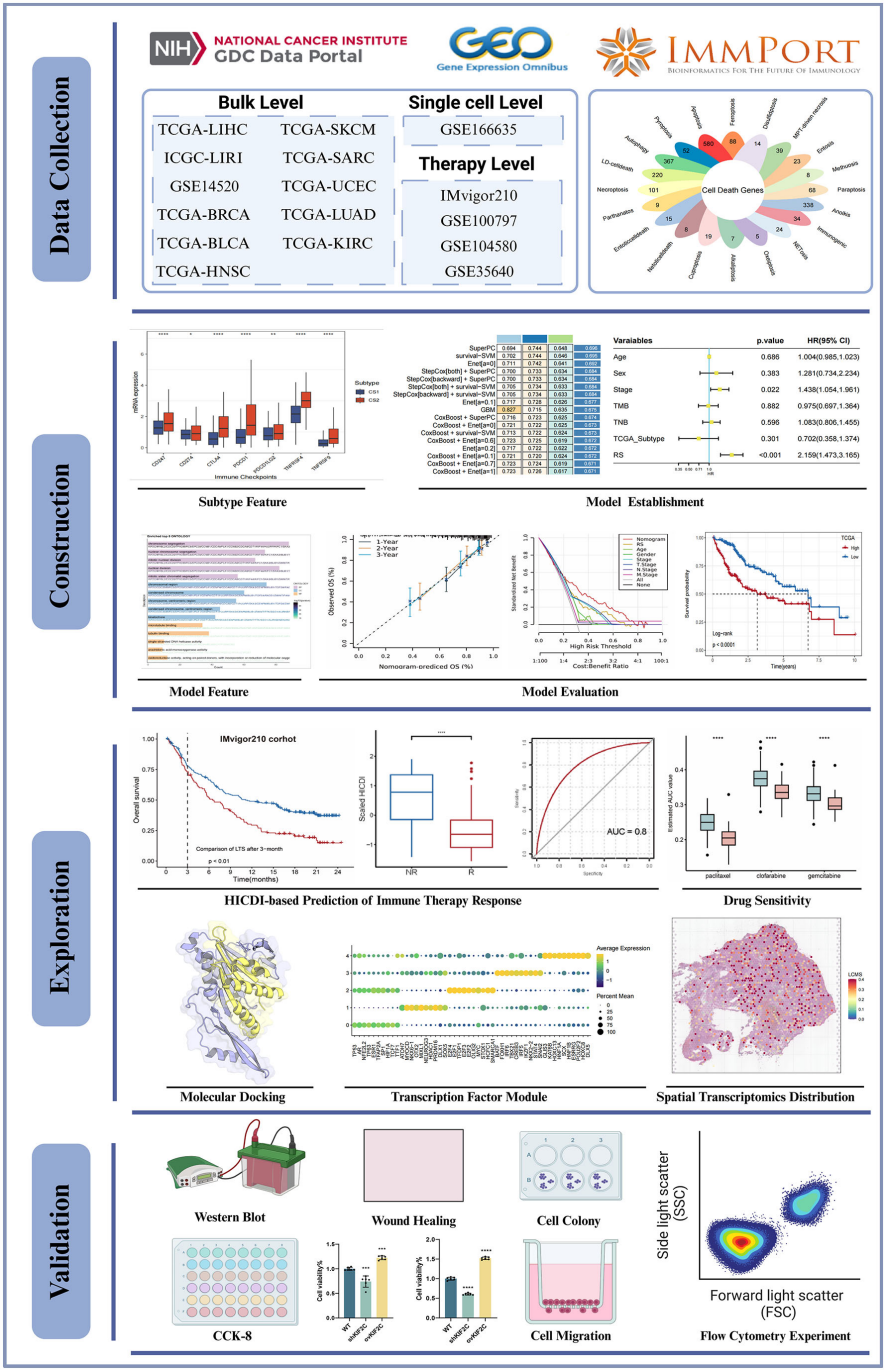
Flowchart of this study.

**Figure 2:**
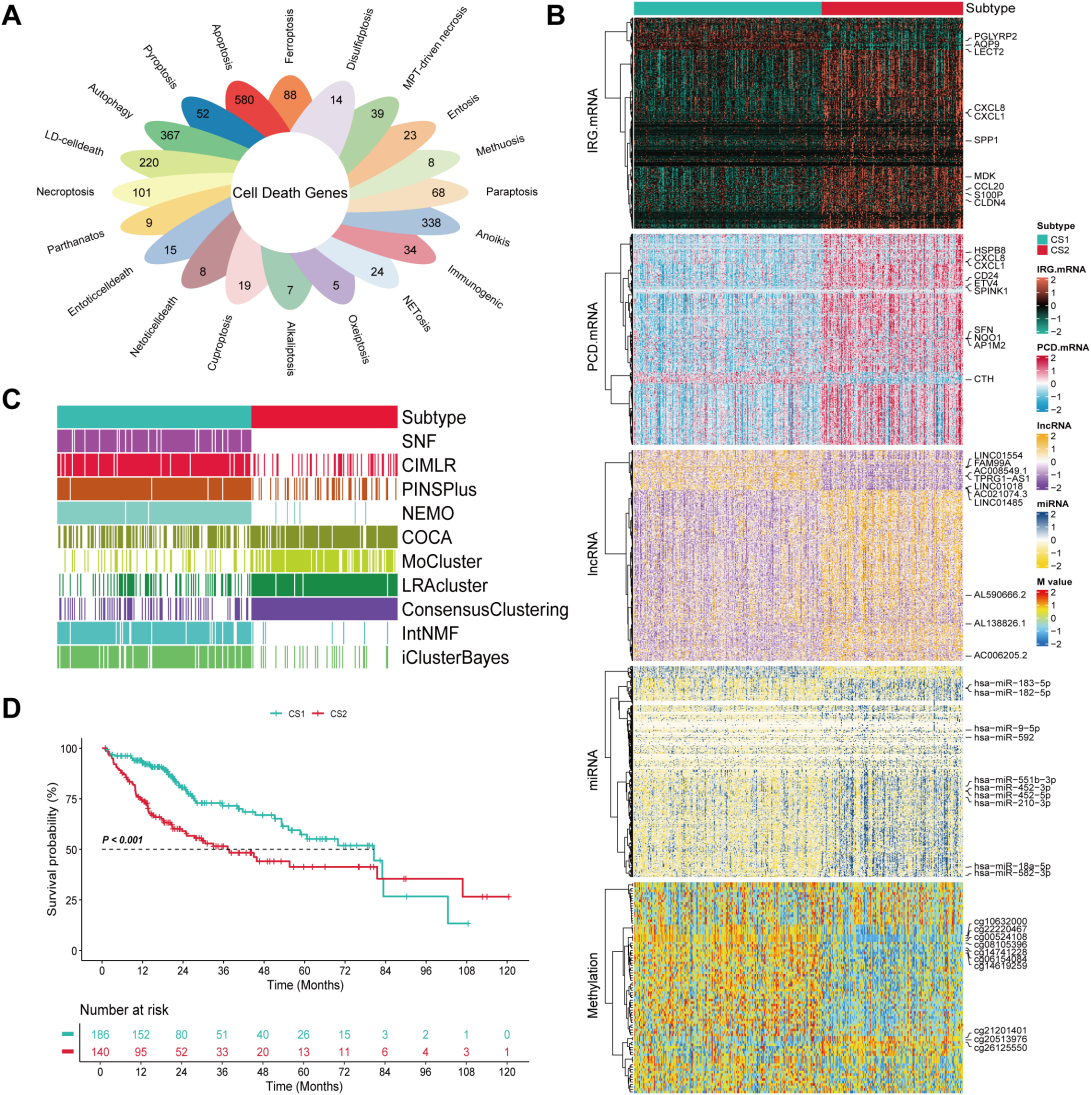
Identification of two key molecular subgroups associated with prognosis in HCC through a multiomics approach. **(A)** The petal diagram displays 20 cell death-related genes. **(B)** Multi-omics characterization, including mRNA, lncRNA, miRNA, and DNA methylation sites. **(C)** Ten multi-omics clustering methods were used to cluster patients with liver cancer. **(D)** Survival outcomes of the two subtypes differ in the TCGA training cohort.

**Figure 1** depicts the technical workflow of this study. We acquired cell death-associated genes from previous studies and included immune-related genes for comprehensive analysis (**Figure 2A**). Subsequently, we correlated immune-related genes, cell death-related genes, lncRNA, miRNA, and methylation data to the TCGA-LIHC cohort after excluding ineligible patients (overall survival [OS] > 30 days) for further analysis (**Figure 2B**). We employed the CPI and gap statistic to determine the ideal number of clusters and selected a k value of 2 for subsequent analysis (**Supplementary Figures 1A–C**). We integrated 10 different algorithms and determined that the molecular subtyping of HCC patients was significantly differentiated when k = 2 (**Figure 2C**). Based on ten algorithms, HCC patients were stratified into two subtypes, CS1 and CS2. Patients in subtype CS2 exhibited a worse prognosis, and those in subtype CS1 exhibited a better prognosis (**Figure 2D**).

To clarify the heterogeneity and clinical significance of tumor molecular subtypes, we identified two subtypes, CS1 and CS2, in TCGA-LIHC based on molecular features. At the genomic level, the two subtypes exhibited significant differences in the FGA and losses/loss of heterozygosity (FGL + FGD) (**Supplementary Figure 2A**). Regarding clinical characteristics, CS1 and CS2 exhibited distinct distributions in tumor stage (**Supplementary Figure 2B**). To investigate the universality and stability of the subtypes, we identified 200 subtype-specific upregulated genes as classifiers based on differential expression analysis and subsequently validated the stability of the subtypes in TCGA-LIHC using NTP and PAM methods. Consistent with this, the alignment of CSs with NTP and PAM algorithms was assessed in two external cohorts, ICGC and GSE14520 (**Supplementary Figures 2C–H**).

### 3.2 Molecular landscape between HCC subtypes

**Figure 3:**
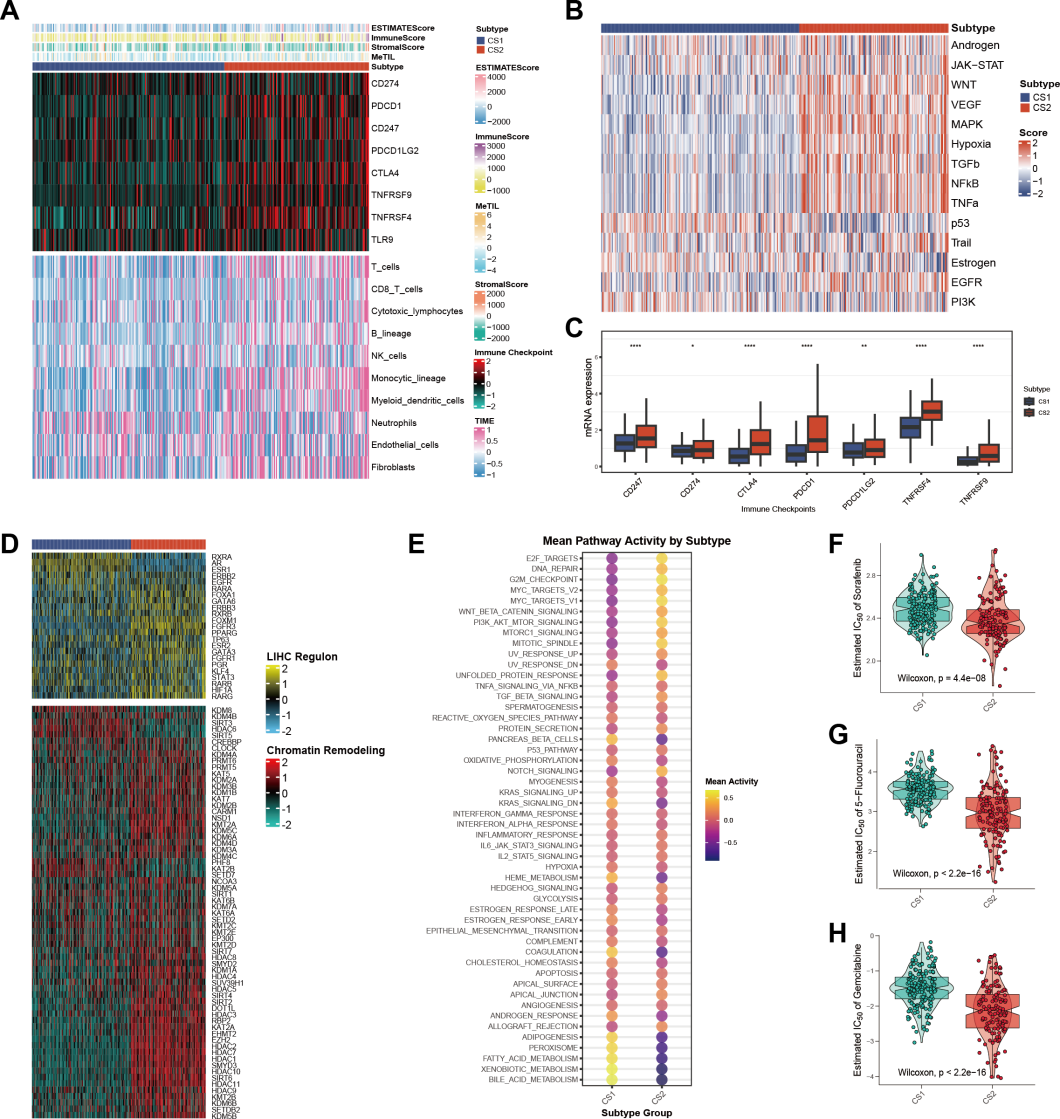
Molecular landscape between HCC subtypes. **(A)** In the TCGA cohort, the heatmap annotations at the top represent immune enrichment scores for tumor-infiltrating lymphocytes, stromal enrichment scores, and DNA methylation-related CTL infiltration. The upper panel indicates the expression of representative immune checkpoint genes, while the lower panel displays the enrichment levels of 10 TME-related immune cells. **(B)** Heatmap and subgroup comparisons of LIHC cancer pathway activity calculated using the PROGENy method. **(C)** Analysis of common immune checkpoint genes across subgroups. **(D)** Regulatory activity profiles of 23 transcription factors (top) and potential regulators involved in chromatin remodeling for the two subtypes (bottom). **(E)** Hallmark gene set scores in different subgroups. **(F, G)** IC_50_ values of commonly used chemotherapeutic agents. *p < 0.05, **p < 0.01, ***p < 0.001.

To analyze immune infiltration of the two subtypes, we assessed the distribution of immune cells and the abundance of immune checkpoint expression across different subtypes. We observed significant heterogeneity in immune-related features between CS1 and CS2 (**Figure 3A**). Subtype CS2 demonstrated higher immune cell infiltration and expression of immune checkpoint molecules, whereas subtype CS1 exhibited comparatively inactive immune response-related features. Subsequently, we examined the disparities in molecular features among the CSs. The results revealed that the activity of common oncogenic pathways differed among different CSs. The Wingless/Integrated, epidermal growth factor receptor, hypoxia, tumor necrosis factor-alpha (TNF-α), nuclear factor kappa-B, and phosphoinositide 3-kinase (PI3K) pathways were significantly more active in subtype CS2, whereas the tumor protein P53 pathway was more active in subtype CS1 (**Figure 3B**).

At the transcriptional level, we reaffirmed that the CS2 subtype has higher expression of immune suppression-related genes (**Figure 3C**). To further examine the transcriptional disparities among subtypes, we assessed putative regulators associated with tumor chromatin remodeling and 23 TFs in LIHC (**Figure 3D**). The regulatory activity of key transcriptional factors in liver cancer, including retinoid X receptor alpha, androgen receptor, ERBB2, and forkhead box A1, exhibited unique clustering patterns among subtypes, displaying subtype-specific differences in the core transcriptional regulatory network. The activity profiles of regulators involved in cancer-associated chromatin remodeling further highlighted the distinct regulatory patterns between the two subtypes. This subtype-specific chromatin remodeling molecular expression pattern may reshape the epigenetic landscape of the genome, drive divergent transcriptional programs, and contribute to the biological heterogeneity observed between subtypes. Concurrently, GSVA analysis based on the HALLMARK gene set delineated the biological characteristics of each subtype (**Figure 3E**). Pathways including epithelial−mesenchymal transition (EMT), hypoxia, G2M checkpoint, and mTORC1 signaling exhibited distinct enrichment patterns between subtypes. To clarify the differences in drug sensitivity between CS1 and CS2 subtypes, we further analyzed their half-inhibitory concentrations for commonly utilized liver cancer drugs, including sorafenib, 5-fluorouracil, and gemcitabine. The results revealed that the CS2 subtype demonstrated markedly greater sensitivity to all three drugs (**Figures 3F–H**). These findings establish a direct pharmacological basis for personalized drug selection in liver cancer according to CS subtyping.

### 3.3 Construction of HCC consensus subtype-specific model genes (HICDI) by developing a competitive machine learning framework

**Figure 4:**
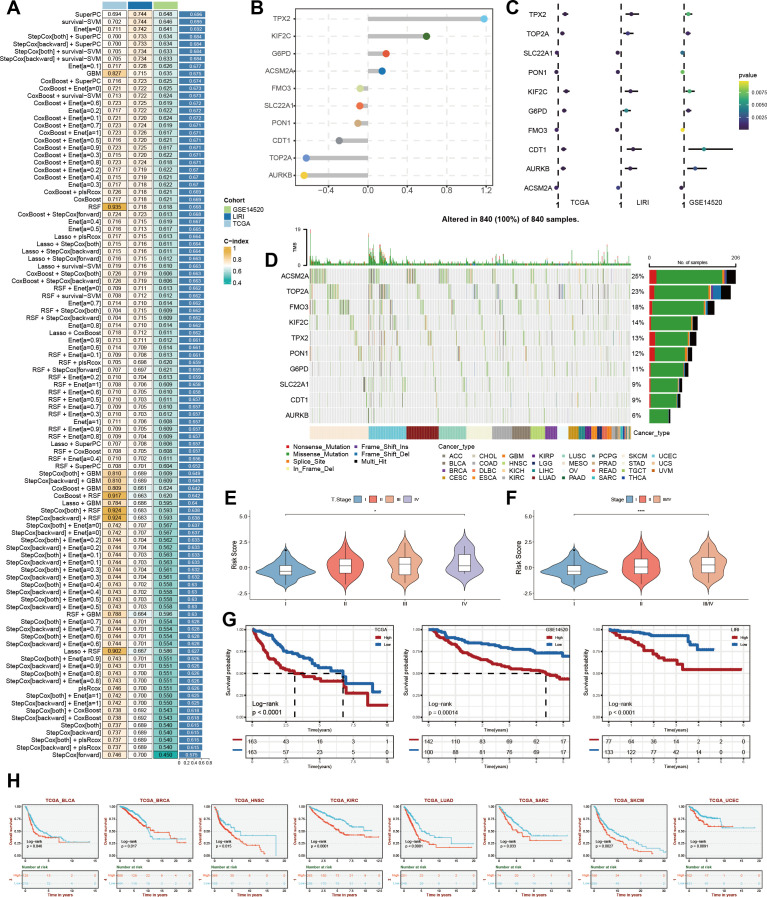
Construction of HICDI by developing a competitive machine learning framework. **(A)** Heatmap illustrating the concordance index (C-index) of the models constructed using 101 machine learning algorithms. **(B)** Hub genes identified using the StepCox algorithm. **(C)** Results of univariate Cox regression analysis for hub genes across different cohorts. **(D)** Genomic alterations of hub genes across multiple cancer types. **(E)** Distribution of T stage according to different HICDI risk groups in the training cohort. **(F)** Distribution of stage according to different risk groups in the training cohort. **(G)** Survival analysis results for different risk groups in the training and validation cohorts. **(H)** Survival analysis results for different risk groups in other cancer types. *p < 0.05, **p < 0.01, ***p < 0.001.

We identified 35 consensus genes significantly associated with OS from immune-cell death subtype-specific genes using univariate Cox regression analysis. Subsequently, using training TCGA-LIHC cohort, we developed a competitive machine learning framework based on these genes and evaluated the performance of each model in two independent validation sets, ICGC and GSE14520. Considering model stability and reliable predictive power, and avoiding the limitations of overfitting and poor interpretability often seen in single models, we selected the model developed by the “StepCox[both] + SuperPC” combination, which achieved an average C-index value of 0.684 in the external datasets (**Figure 4A**). We identified 10 hub genes utilizing the StepCox [both] algorithm (**Figures 4B, C**). Subsequently, we examined the prevalence of these 10 hub genes across pancancer genomes and discovered that among the identified key genes, ACSM2A (25%), TOP2A (23%), and FMO3 (18%) exhibited elevated mutation frequencies. The mutation types included missense mutations, nonsense mutations, splice site mutations, and others (**Figure 4D**). Additionally, we employed K-M analysis, and the findings were predominantly consistent with those obtained using the Cox algorithm (**Supplementary Figure 3**). Subsequently, utilizing the GSCALite platform, we identified a positive correlation between mRNA expression levels and copy number variations (CNVs) of HICDI genes across most cancer types, notably for TPX2 (**Supplementary Figure 4A**). The methylation levels of HICDI genes were negatively correlated with mRNA expression levels in most cancer types, with reduced methylation levels observed in tumor tissues (**Supplementary Figures 4B, C**). These findings indicate that HICDI genes may affect patient prognosis through epigenetic alterations. Furthermore, we observed that essential genes, including KIF2C, AURKB, and CDT1, exhibited significant activation in pathways that facilitate tumor progression, including cell cycle, DNA damage response, and epithelial-mesenchymal transition (**Supplementary Figure 4D**).

To investigate the correlation between the model risk score and clinical staging, we conducted a detailed analysis of the distribution characteristics of the risk score across various T stages and overall stages. The results revealed that the model risk score correlated significantly with tumor clinical staging, with higher risk scores associated with more advanced stages (**Figures 4E, F**). Furthermore, we calculated the HICDI score for each sample across all cohorts. Patients with high HICDI scores demonstrated poorer clinical outcomes in TCGA-LIHC, ICGC, and GSE14520 (**Figure 4G**). To assess the generalizability of the model, we utilized the model genes to evaluate multiple cancer types using the GSVA algorithm and performed prognostic analyses. We found that patients in the high-score group typically exhibited worse prognoses (**Figure 4H**). These findings offer vital insights for prognostic stratification and mechanism investigation across various cancer types.

### 3.4 Comparison of HICDI with other models

**Figure 5:**
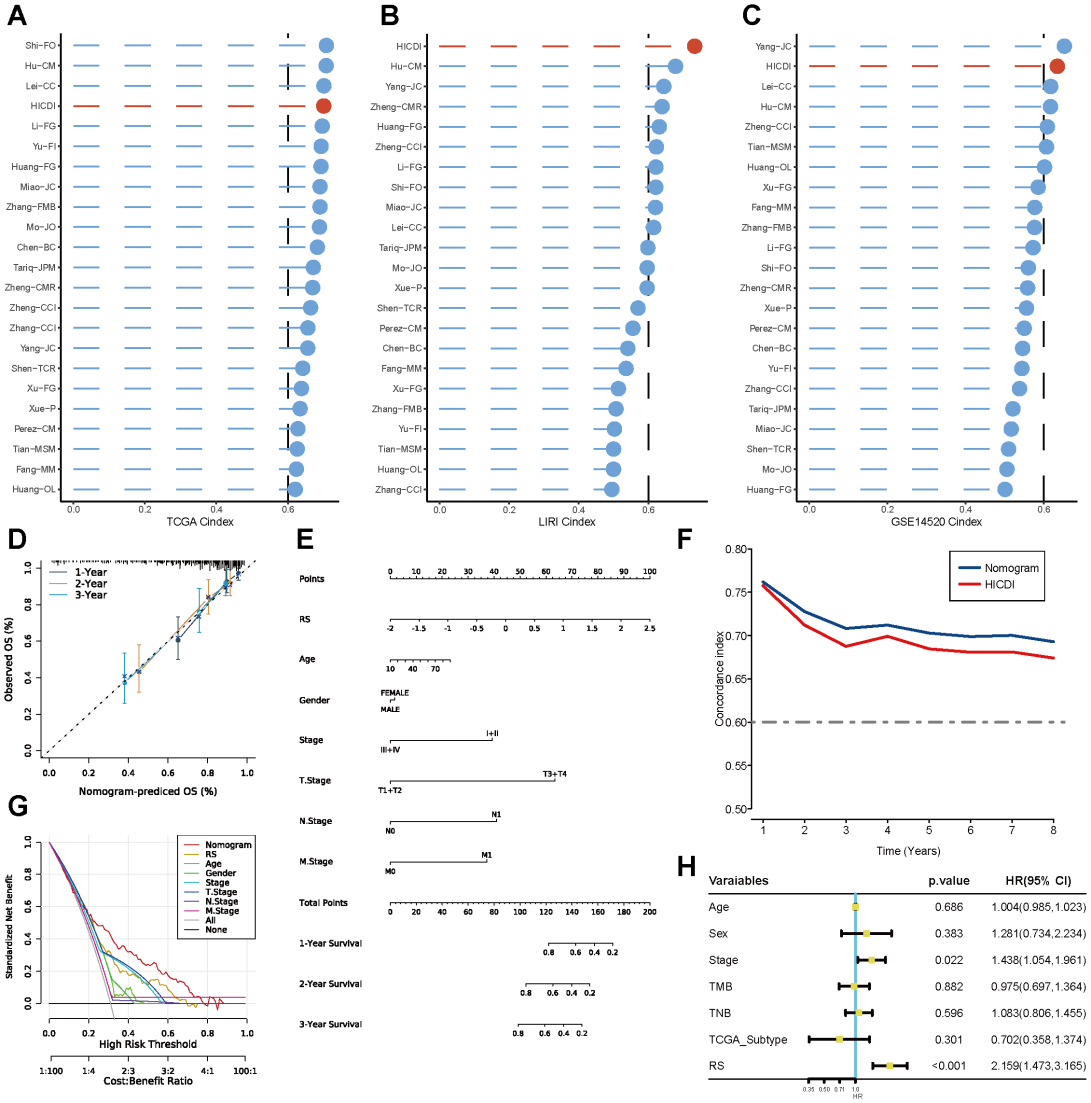
Comparison of HICDI with other models. **(A–C)** Comparison of HICDI performance with previously published models in TCGA-LIHC, ICGC-LIRI, and GSE14520 cohorts. **(D)** Calibration curve of the comprehensive nomogram. **(E)** Comprehensive nomogram based on risk scores. **(F)** Comparison of the time-dependent C-index of the comprehensive nomogram and HICDI. **(G)** Decision curve analysis of the comprehensive nomogram. **(H)** Multivariate analysis of risk scores and other clinical indicators to determine the value of risk scores.

Recent advancements in high-throughput sequencing technology have driven groundbreaking progress in oncology, enabling the development of stratified treatment strategies and the implementation of precise, individualized therapies for patients (35). Multiple studies have been conducted to develop prognostic features based on machine learning methods to predict cancer outcomes (36). To facilitate a comprehensive comparison between HICDI and other models, we conducted a systematic review of relevant literature published in the past five years, including 22 different models in our study. Notably, HICDI exhibited excellent performance in TCGA-LIHC, ICGC, and GSE14520 datasets, achieving good C-index performance (**Figures 5A–C**).

We constructed a detailed nomogram that integrates clinical prognostic factors. The calibration curve indicated that the nomogram has accuracy consistent with actual outcomes. Decision curve analysis (DCA) revealed that the nomogram offers significantly greater clinical benefit to patients than the HICDI index alone. Furthermore, the time-dependent C-index confirmed that the nomogram exhibits superior predictive performance (**Figures 5D–F**). Subsequently, we identified the risk score as an independent prognostic factor for poor outcomes in patients with HCC using multivariate Cox analysis (**Figure 5H**).

### 3.5 Immunological characteristics of HICDI

**Figure 6:**
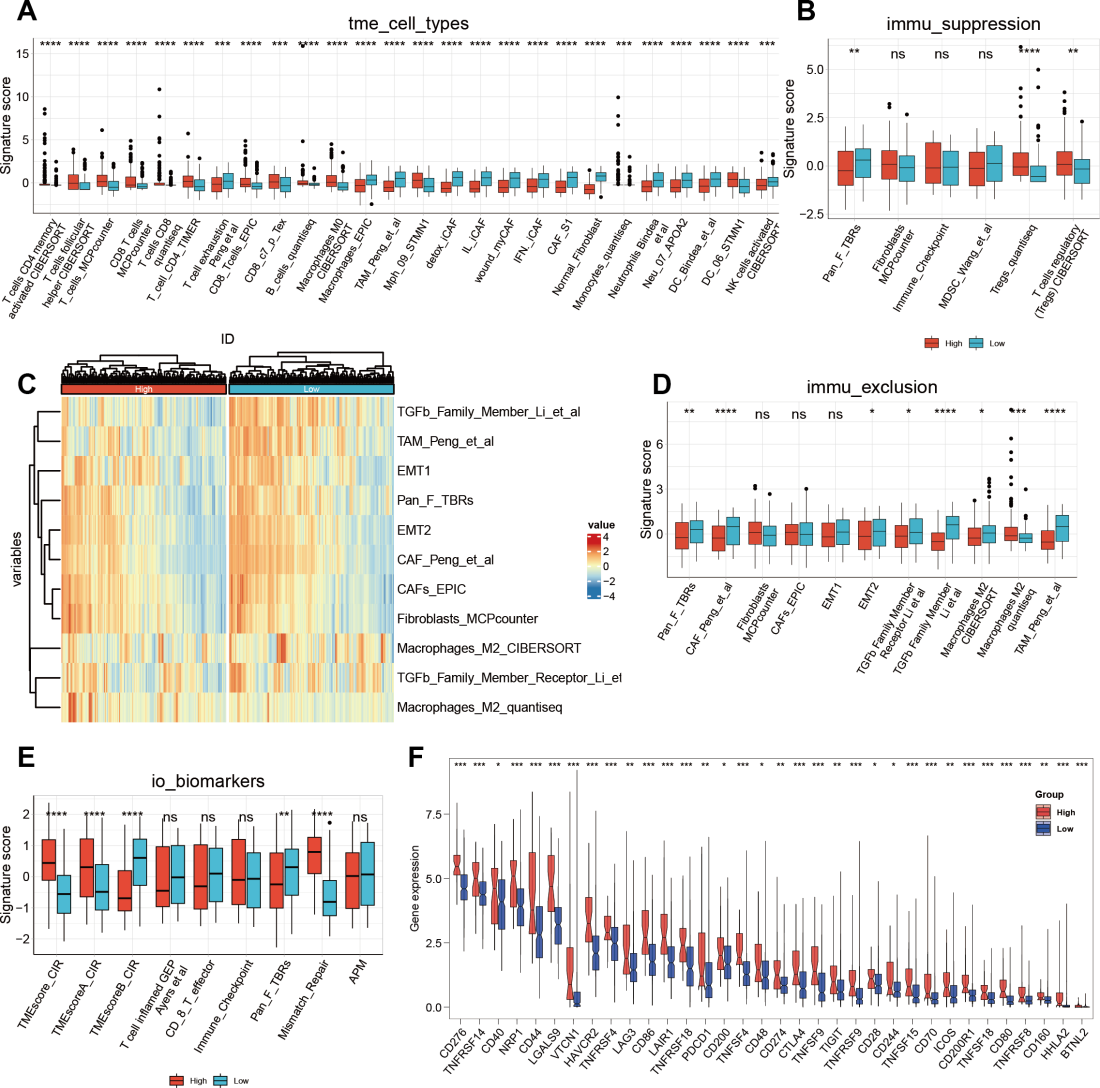
Immune characteristics of HICDI. **(A)** Differences in TME immune cell type characteristics distribution between patients with high and low HICDI. **(B)** Differences in TME immune suppression characteristics distribution between patients with high and low HICDI. **(C-D)** Differences in TME immune exhaustion characteristics distribution between patients with high and low HICDI. **(E)** Differences in the distribution of biomarker characteristics between patients with high and low HICDI. **(F)** Differences in immune checkpoint distribution between patients with high and low HICDI. *p < 0.05, **p < 0.01, ***p < 0.001.

To systematically analyze the TME characteristics of LIHC and their correlation with HICDI, we employed the IOBR R package for comprehensive TME immune infiltration assessment. We observed significant differences in the infiltration of activated CD4 memory T cells, helper T cells, CD8 T cells, macrophages, and other immune cells between high- and low-risk groups, highlighting substantial heterogeneity in the immune microenvironment between the two groups (**Figure 6A**). Furthermore, we observed that regulatory T cells (Tregs), associated with immune suppression, were markedly enriched in the high-risk group (**Figure 6B**). Tregs, as core immunosuppressive cells, can establish an immunosuppressive TME by secreting inhibitory cytokines and directly suppressing the function of effector immune cells through cell-to-cell contact (37–39). Subsequently, we examined immune fatigue-related indicators in both groups and discovered that the low-risk group exhibited relatively higher expression of immune exhaustion markers yet demonstrated better prognosis (**Figures 6C, D**). Based on the previous results, we believe that the low-risk group is more likely characterized by immune activation accompanied by exhaustion, which can maintain a certain level of antitumor immunity. This indicates that low HICDI HCC is more likely to be a “cold tumor.” Signatures previously identified as correlating with improved immunotherapy response were also significantly enriched in the high HICDI group (**Figure 6E**). Subsequently, we examined the expression of immune suppression-related genes in the high- and low-risk groups and observed higher expression of these genes in the high-risk group (**Figure 6F**).

To evaluate the immune response in different risk groups, we employed the TIDE algorithm for a comprehensive assessment of the immune response across various risk groups. The results revealed that the high-risk group exhibited a significantly higher TIDE composite score compared to the low-risk group, along with stronger immune exhaustion and pro-inflammatory cytokine expression (**Supplementary Figures 5A–D**). Subsequently, we examined the molecular characteristics of the two groups through Hallmark core pathway analysis and observed that the high-risk group significantly activated pathways associated with cell cycle regulation, proliferation, and growth signaling (PI3K-AKT), DNA repair, and glycolysis (**Supplementary Figure 5E**). However, pathways associated with energy homeostasis and metabolic breakdown, including oxidative phosphorylation, fatty acid metabolism, and bile acid metabolism, were relatively inhibited in the high-risk group. GO analysis indicated that biological processes, including chromosome segregation, nuclear chromosome segregation, and mitotic nuclear division, were significantly enriched in the high HICDI expression group, which are integral in the core steps of cell cycle regulation and cell proliferation (**Supplementary Figure 5F**). Tumor mutation burden (TMB) and tumor neoantigen burden (TNB) are recognized biomarkers for assessing patient response to immunotherapy. Consequently, we examined the disparities in these biomarkers between the two groups. The high HICDI expression group had higher TMB and TNB, suggesting that this group may have enhanced immunogenicity (**Supplementary Figures 5G, H**).

### 3.6 The value of HICDI in predicting immunotherapy response

**Figure 7:**
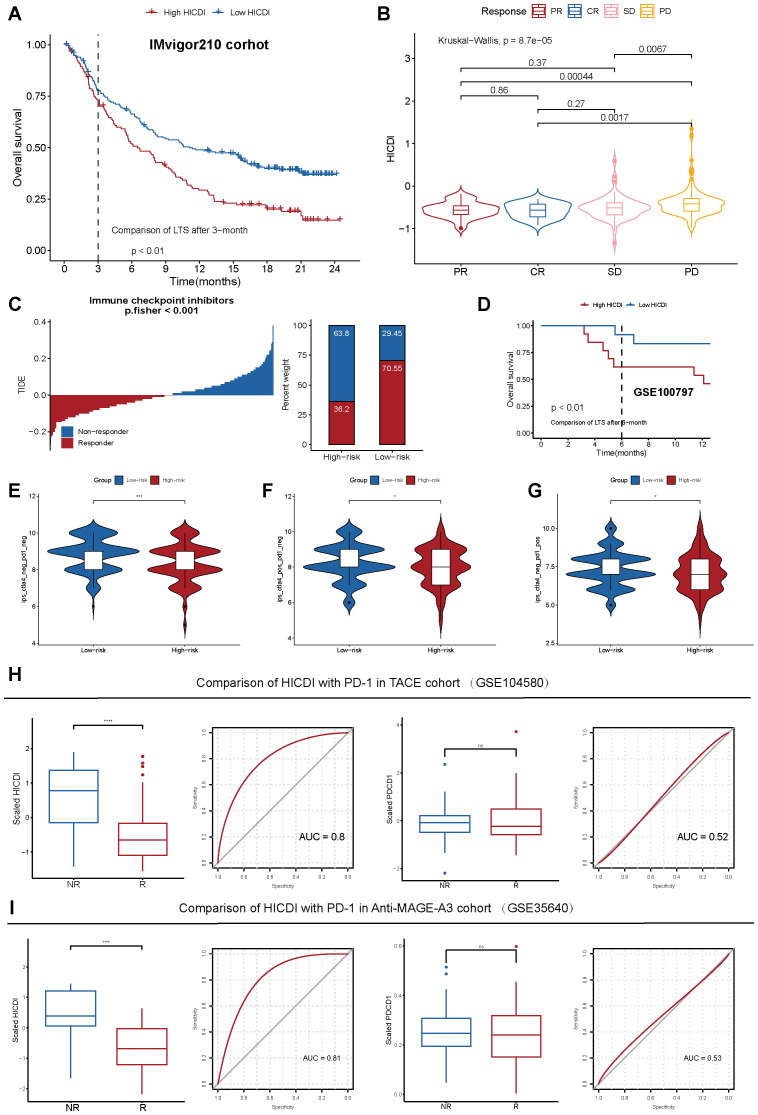
HICDI demonstrates excellent predictive capability for immunotherapy response. **(A)** Differences in LTS after 3 months of treatment between high and low HICDI groups in the IMvigor210 cohort. **(B)** Distribution of HICDI in different immunotherapy response groups. **(C)** Prediction of immunotherapy response between high and low HICDI groups using the TIDE algorithm. **(D)** Differences in LTS after 3 months of treatment between high and low HICDI groups in the GSE100797 cohort. **(E–G)** IPS scores for high and low HICDI groups. **(H)** IPS scores for high and low HICDI groups. **(H)** Box plots and ROC curves illustrate the performance of HICDI (left panel) and PD-1 (right panel) in predicting TACE efficacy in the GSE104580 cohort. **(I)** Box plots and ROC curves illustrate the performance of HICDI (left panel) and PD-1 (right panel) in predicting anti-MAGE-A3 efficacy in the GSE35640 cohort. *p < 0.05, **p < 0.01, ***p < 0.001.

We conducted a thorough analysis to comprehensively evaluate the role of HICDI in immunotherapy. Initially, we examined the IMvigor210 cohort and identified significant differences in long-term survival (LTS) between patients with high and low HICDI scores three months post-treatment (p < 0.01), with better LTS in the low HICDI score group, demonstrating that this group benefited more from the treatment (**Figure 7A**). Furthermore, we found that in the IMvigor210 cohort, the HICDI score was markedly lower in the responder group (complete response [CR]/partial response [PR]) compared to the non-responder group (progressive disease [PD]/stable disease [SD]) (**Figure 7B**). Subsequently, we evaluated the patients’ response to immunotherapy using the TIDE algorithm and observed that the low HICDI score group exhibited better responsiveness (**Figure 7C**). Additionally, in the T-cell therapy cohort (GSE100797), we observed that patients in the high HICDI score group had worse LTS (**Figure 7D**). To comprehensively evaluate the role of HICDI in HCC immunotherapy, we examined the immunophenoscore (IPS) scores derived from the TCIA database. A higher IPS score signifies a better response to ICI treatment, including PD-1 inhibitors and CTLA4 inhibitors. We classified them into four groups: ips_ctla4_pos_pd1_pos, ips_ctla4_pos_pd1_neg, ips_ctla4_neg_pd1_pos, and ips_ctla4_neg_pd1_neg. The results revealed that patients in the low HICDI score group exhibited better responses to anti-PD-1 and anti-CTLA4 treatments (**Figures 7E–G**).

In the TACE-treated cohort (GSE104580), we assessed the prediction accuracy of the HICDI by comparing it with the consensus biomarker PD-1. The results revealed that HICDI effectively demonstrated therapeutic efficacy, whereas PD-1 exhibited poor predictive performance (**Figure 7H**). In the GSE35640 cohort receiving anti-MAGE-A3 immunotherapy, HICDI surpassed PD-1 (**Figure 7I**). These findings suggest that most conventional markers perform well only under specific conditions. Compared to typical biomarkers, HICDI exhibits a favorable performance advantage, providing preliminary exploratory evidence for subsequent clinical translation and application.

### 3.7 Screening of potential therapeutic drugs

**Figure 8:**
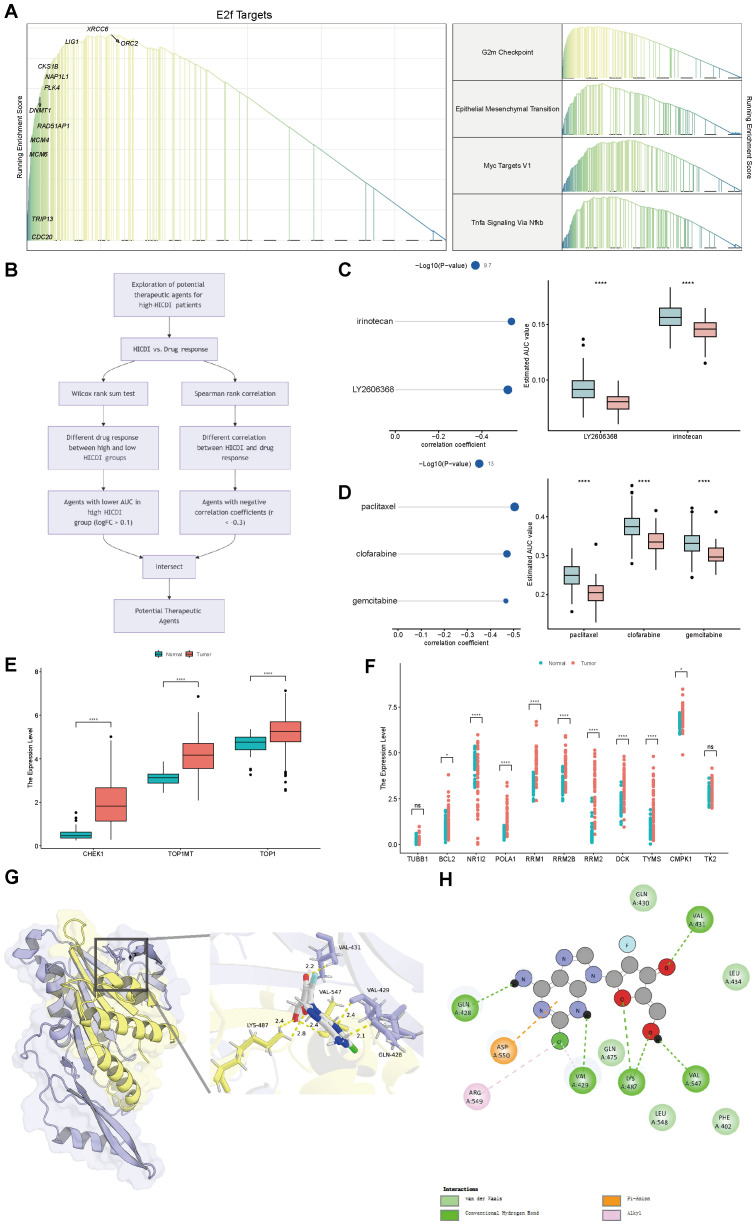
Screening of potential therapeutic drugs. **(A)** GSEA analysis reveals significant activation of oncogenic pathways in patients with high HICDI. **(B)** Comprehensive screening process for potential drugs. **(C)** Analysis of correlation and group differences for drugs identified from the PRISM database. **(D)** Analysis of correlation and group differences for drugs identified from the CTRP database. **(E, F)** Analysis of group differences for potential drug target genes. **(G, H)** Display of molecular docking results between the target protein KIF2C and clofarabine. *p < 0.05, **p < 0.01, ***p < 0.001.

Patients with high and low HICDI scores exhibited significant prognostic differences. GSEA analysis revealed that pathways such as E2F signaling, EMT, cell cycle, and proliferation were significantly activated in patients with high HICDI scores (**Figure 8A**). Subsequently, we utilized CTRP and PRISM to identify possible therapeutic agents for patients with high HICDI scores. We systematically investigated potential drugs for patients with high HICDI (**Figure 8B**). We identified two PRISM drugs, LY2606368, and three CTRP drugs, paclitaxel, clofarabine, and gemcitabine (**Figures 8C, D**). Drugs such as paclitaxel and gemcitabine have already been studied and experimentally confirmed in HCC treatment research (40–43). Subsequently, we evaluated the differences in the target genes of the candidate drugs between tumor and normal tissues (**Figures 8E, F**). The higher expression levels of the target genes in tumor tissues confirm the therapeutic potential of the candidate drugs.

Multiple studies have extensively investigated the functions of prexasertib, gemcitabine, paclitaxel, and irinotecan in HCC treatment (44–47). Clofarabine, a second-generation antitumor drug, has exhibited significant anticancer efficacy, especially in the management of acute lymphoblastic leukemia, and has been proven to possess radiosensitizing effects. However, research on the utilization of clofarabine for HCC treatment remains relatively scarce. Therefore, we performed molecular docking analysis of clofarabine and the protein encoded by the HICDI core gene KIF2C utilizing the CB-Dock platform. The results revealed that the absolute value of the Vina Score was 6.8kcal/mol, indicating that clofarabine can form a good binding interaction with the KIF2C protein (**Figures 8G, H**).

### 3.8 Integration of single-cell and spatial transcriptomics profoundly elucidates the role patterns of HICDI within the microenvironment

To investigate HICDI distribution in various cell types within the TME, we performed single-cell analysis on two primary HCC cases from the GSE166635 cohort. Using canonical marker genes for different cell types, we identified seven major clusters: fibroblasts, myeloid cells, endothelial cells, hepatocytes, malignant cells, NK/T cells, and B cells (**Supplementary Figures 6A, B**). Subsequently, we examined the HICDI gene distribution within the TME and observed that most HICDI genes were highly expressed in certain malignant and myeloid cells (**Supplementary Figure 6C**). We employed the AUCell algorithm to score HICDI at the cellular level, which similarly revealed high HICDI scores in some malignant and myeloid cells (**Supplementary Figure 6D**). Given our earlier observation of significant activation of cell cycle and proliferation pathways in the high HICDI score group, we further examined MKI67 distribution and, as expected, found that MKI67 was highly localized within specific malignant and selected myeloid cell subsets within the TME (**Supplementary Figure 6E**). Based on these observations, we further analyzed the co-expression of HICDI and MKI67 at the single-cell level and observed a significant positive correlation between the two in malignant and myeloid cells (**Supplementary Figures 6F, G**).

**Figure 9:**
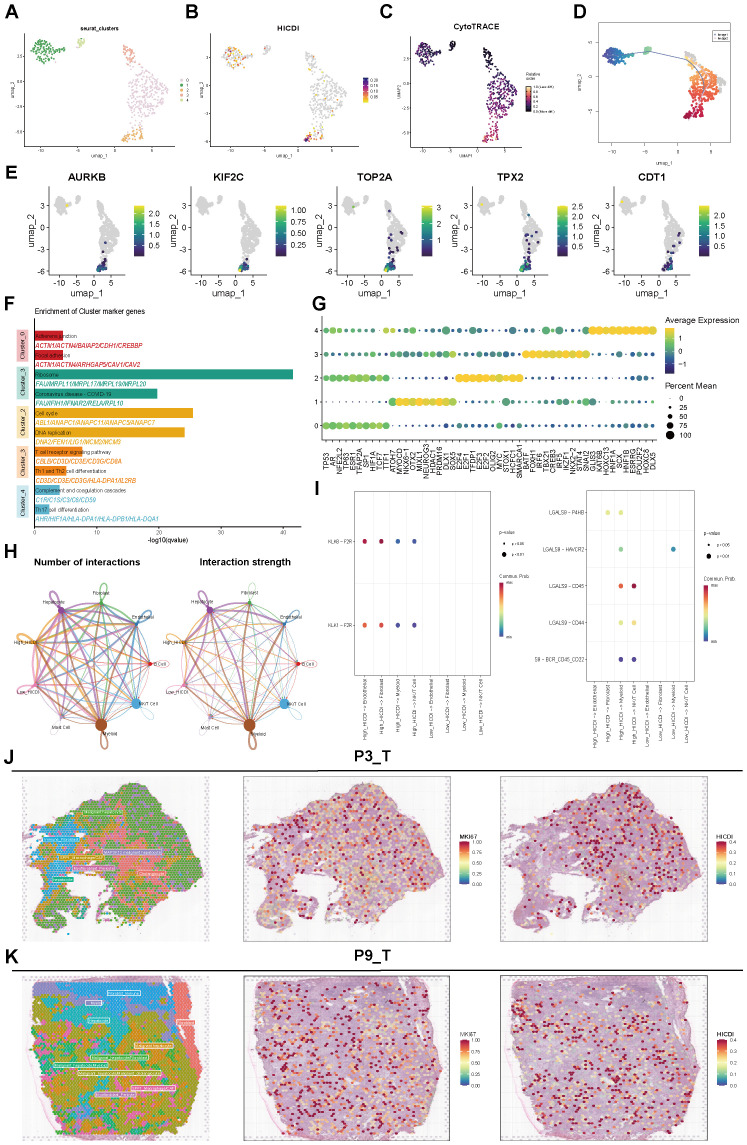
Integration of single cell and spatial transcriptomics significantly elucidates the role patterns of HICDI within the microenvironment. **(A)** Illustration of malignant cell clustering. **(B)** Distribution of HICDI in malignant cells. **(C)** DCytotrace assessment of cellular differentiation potential. **(D)** Singshot analysis of malignant cell developmental trajectories. **(E)** Expression of HICDI-related genes in malignant cells. **(F)** Pathway enrichment analysis of malignant cell clusters. **(G)** Analysis of the top 10 transcription factors in malignant cell clusters. **(H)** Number and weight of cell-cell interaction networks inferred by CellChat. **(I)** Interaction network diagram of KLK and GALECTIN signals inferred using CellChat. **(J, K)** Spatial transcriptomics analysis of HICDI and MKI67 distribution.

To further investigate the features of malignant cells, we isolated the malignant cell population and re-clustered it at an appropriate resolution, resulting in five clusters (**Figure 9A**). Subsequently, we analyzed the distribution of HICDI among the malignant cell population and discovered that they were predominantly concentrated in cluster 2 of the malignant cell population (**Figure 9B**). Utilizing the cytotrace algorithm, we observed that cluster 2 exhibited a relatively lower degree of differentiation, signifying higher stemness and proliferative potential (**Figure 9C**). Furthermore, we utilized Slingshot to examine the differentiation of malignant cells and observed that in the GSE166635 dataset, cluster 2 functioned predominantly as the starting cluster and differentiated into two lineages (**Figure 9D**). Additionally, analysis revealed that some HICDI genes were highly expressed in cluster 2 (**Figure 9E**). Subsequently, pathway enrichment analysis of each cell cluster revealed that cluster 2 was predominantly enriched in the cell cycle and DNA replication pathways (**Figure 9F**). We utilized decoupleR to analyze the activity of the top 10 contributing transcription factors to investigate the transcriptional regulatory network heterogeneity among different cell clusters (**Figure 9G**). To investigate the potential mechanisms underlying high HICDI score malignant cells, we employed the CellChat tool to examine their intercellular interactions with other components within TME. Our findings indicate that high HICDI score malignant cells exhibited significantly enhanced communication strength and frequency with myeloid cells and NK/T cells compared to low HICDI score malignant cells (**Figure 9H**). Subsequent findings revealed that high HICDI score malignant cells may modulate stromal and immunological cells through the kallikrein-related peptidase (KLK) and GALECTIN signaling pathways, enhancing the development of an immunosuppressive microenvironment (**Figure 9I**). The results revealed that in cluster 2, these transcription factors were significantly associated with the regulation of cell cycle and proliferation pathways. We examined the distribution of HICDI among the spatial transcriptomics cohort. The findings demonstrated that HICDI was predominantly localized in malignant cells and co-expressed with MKI67 (**Figures 9J, K**).

### 3.9 The oncogenic role of KIF2C in hepatocellular carcinoma

**Figure 10:**
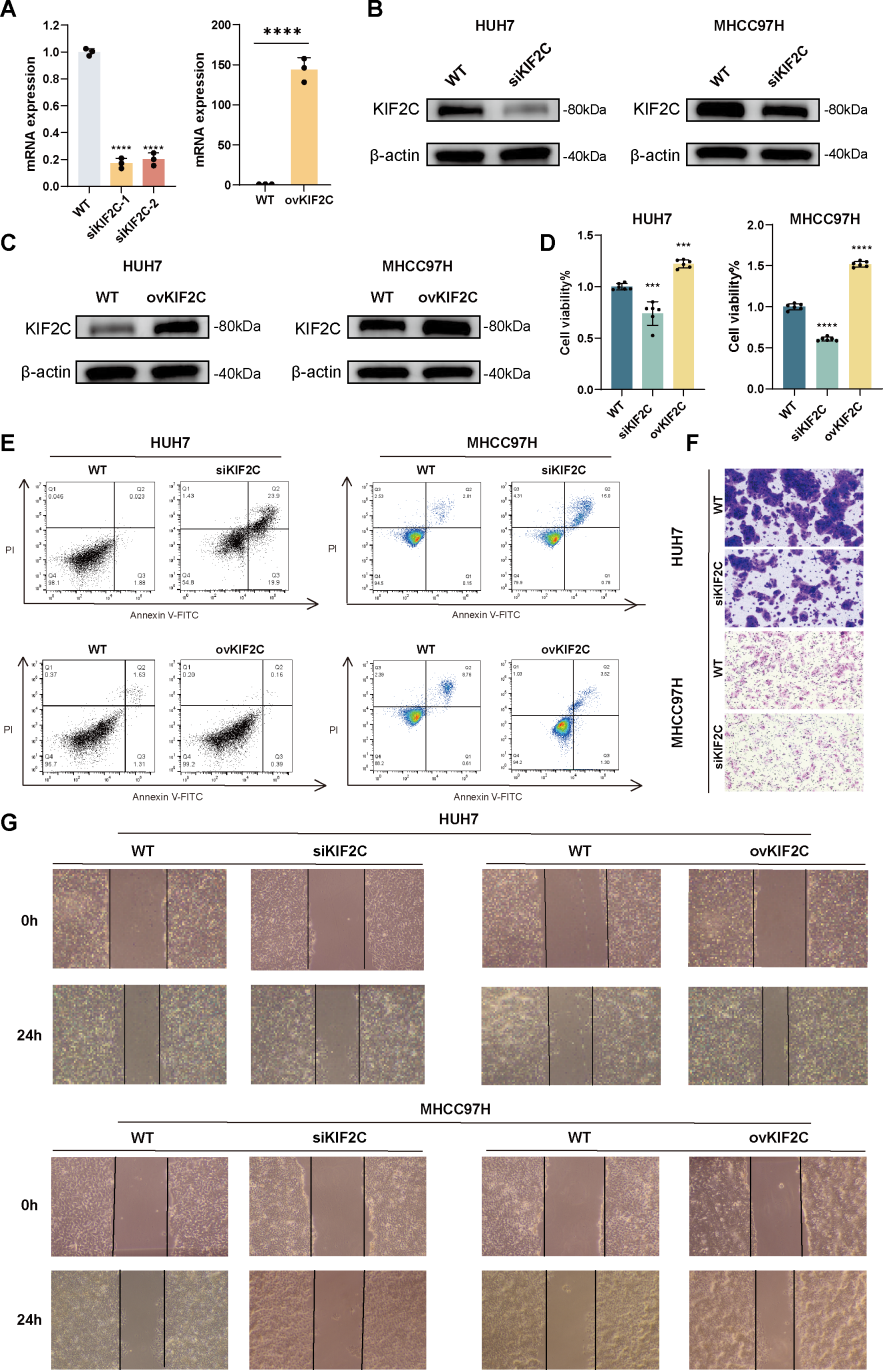
The oncogenic role of KIF2C in hepatocellular carcinoma. **(A–C)** Validation of KIF2C knockdown and overexpression at the RNA and protein levels in HUH7 and MHCC97H cell lines. **(D)** Determination of relative cell viability in HUH7 and MHCC97H cells at 48 h post KIF2C knockdown and overexpression(n=3). **(E)** Detection of cell apoptosis in HUH7 and MHCC97H cells following KIF2C knockdown and overexpression(n=3). **(F)** Invasive capacity of KIF2C-knockdown HUH7 and MHCC97H cells(n=3). **(G)** Migration capacity of HUH7 and MHCC97H cell lines(n=3). *p < 0.05, **p < 0.01, ***p < 0.001.

In subsequent studies, we aimed to identify potential therapeutic targets for hepatocellular carcinoma (HCC) among the 10 HICDI genes. We first investigated the impact of these genes on patients’ overall survival (OS) at the gene expression level. The results demonstrated that all these genes exerted a significant effect on OS, and based on the analytical results of the present study, the KIF2C gene exhibited particularly prominent effects. Previous researchers have explored the influence of KIF2C overexpression on the survival outcomes of HCC patients using bioinformatics approaches (48); however, robust experimental evidence in this regard remains scarce.Given the aforementioned research background, we conducted preliminary experimental investigations into the role of KIF2C in the initiation and progression of HCC. First, we selected two commonly used HCC cell lines (HUH7 and MHCC97H) and established KIF2C knockdown and overexpression cell models using two independent small interfering RNA (siRNA) sequences and one overexpression plasmid, respectively (**Figures 10A–C**). Previous studies have demonstrated that KIF2C can promote tumor cell proliferation in breast cancer. Consistently, in the present study, KIF2C knockdown significantly inhibited the proliferative capacity of HCC cells, whereas KIF2C overexpression enhanced cell proliferation (**Figure 10D**). The EdU incorporation assay also confirmed that KIF2C knockdown suppressed cell proliferation (**Supplementary Figure 7A**). Furthermore, KIF2C knockdown increased the apoptotic rate of HCC cells, whereas KIF2C overexpression reduced the apoptotic level (**Figure 10E**). In addition, results from the colony formation assay revealed that the proliferative activity of HCC cells was markedly impaired following KIF2C knockdown (**Supplementary Figure 7B**). The migratory capacity of HCC cells exhibited a consistent trend: KIF2C knockdown decreased cell migration, while KIF2C overexpression significantly enhanced this capability (**Figure 10F**). Subsequent cell invasion assays further verified that KIF2C knockdown downregulated both the migratory and invasive capacities of HCC cells (**Figure 10G**).

In conclusion, KIF2C is likely to play a crucial role in the progression and immune infiltration of HCC.

## 4 Discussion

HCC is a malignant tumor that poses a severe threat to human health globally (49, 50). Although current clinical treatment of HCC has developed into a multi-modal intervention system, including local curative approaches such as surgical resection and percutaneous ablation (radiofrequency ablation and microwave ablation), targeted drug therapies such as sorafenib and lenvatinib, and immunotherapies represented by PD-1/PD-L1 inhibitors (nivolumab and pembrolizumab), which have provided new treatment options for advanced disease (51–53), the prognosis of patients with advanced HCC remains poor, with a 5-year survival rate of less than 15% (54). These outcomes highlight the limitations of current treatment strategies and underscore the unmet clinical needs for improved treatment modalities. Insufficient understanding of the immune regulation mechanisms within the HCC TME and the lack of personalized treatment strategies are key issues that need attention. Therefore, elucidating potential molecular mechanisms and identifying new prognostic biomarkers could facilitate precision and personalized treatment for patients with cancer (55). Further in-depth investigation of mechanisms will facilitate the discovery and development of new treatment strategies for HCC (56).

Herein, we focused on the key biological processes in HCC that influence disease progression and treatment response and selected 20 cell death-related and immune-related genes as core analysis targets. To comprehensively analyze the expression patterns and clinical relevance of these genes in HCC, we integrated multi-omics data, including immune gene expression profiles, cell death-related gene expression profiles, lncRNA expression profiles, miRNA regulatory networks, and whole-genome DNA methylation data. We utilized ten clustering algorithms for joint analysis and classification of patients with HCC into two molecular subtypes (CS1 and CS2) with significant prognostic outcomes. Survival analysis revealed that patients in the CS1 group exhibited significantly longer overall survival than those in the CS2 group. This subtype classification was robustly validated in the independent external validation datasets GSE14520 and ICGC, confirming its reliability and generalizability.

Analysis of the molecular characteristics and immune infiltration of the two subtypes (CS1 and CS2) revealed that although the CS2 subtype exhibited higher immune cell infiltration, it simultaneously exhibited increased expression of immune suppression-related markers. This paradoxical “high-infiltration yet high-suppression” phenotype may underlie their reduced responsiveness to immunotherapy. Regarding molecular characteristics, the CS2 subtype was enriched for cell cycle-related, proliferation-associated, and DNA repair pathways, and other oncogenic pathways. The abnormal activation of these pathways supports the rapid tumor cell proliferation and genomic instability and is strongly associated with therapeutic resistance. The high immunosuppressive TME with a high proliferation-high repair molecular phenotype of the CS2 subtype together constitutes the core mechanistic basis for its poor prognosis and treatment resistance. This finding provides a mechanistic explanation for the prognostic differences between CS1 and CS2 subtypes and has direct implications for clinical treatment strategy selection. For patients with the CS2 subtype, monotherapy with immunotherapy or targeted therapy is unlikely to overcome its intrinsic resistance barriers. Instead, combination strategies employing immune modulators alongside pathway inhibitors may be more effective in simultaneously reversing immune suppression while simultaneously restricting tumor cell proliferation and repair pathways, achieving precise therapeutic intervention (57–59).

Machine learning algorithms are now widely utilized in clinical prediction (60, 61). To clarify the differences in molecular characteristics between different prognostic subtypes and enhance clinical utility, we selected the optimal model HICDI through 101 algorithm combinations to overcome the limitations associated with algorithm selection. Previous studies have proposed that extracting stable latent features while effectively suppressing interfering variations corroborates the necessity and stability of the HICDI framework in deriving a stable immune-cell death signature from the highly heterogeneous multi-omics signals of hepatocellular carcinoma (62). The predictive performance of the HICDI model is largely comparable to that of previously published prognostic models for HCC, and exhibits a modest predictive advantage in certain validation cohorts. Furthermore, we observed that across multiple cancer types, patients in the high HICDI score group consistently showed a trend toward poorer prognosis, a finding that provides preliminary exploratory evidence to support the potential generalizable application of this integrated model. To further elucidate the molecular and immunological feature mining for high- and low- HICDI risk groups and identify potential targets. Our results revealed that the high HICDI group is significantly associated with proliferation-related biological processes, including DNA replication and cell cycle, whereas the low HICDI group is primarily related to some metabolic pathways. These findings provide some insights into the worse prognosis observed in the high HICDI group. Regarding immune characteristics, although the high HICDI group exhibited higher levels of immune cell infiltration, key immunosuppressive markers, including PD-1, CTLA-4, and immunosuppressive cytokine INFγ, are markedly upregulated, indicating that the TME of patients in the high HICDI group is in an immunosuppressive state, in which the antitumor activity of infiltrating immune cells is severely impaired. These findings indicate that the HICDI model can effectively predict the prognosis of patients with HCC and serve as a valuable tool for guiding treatment decisions. For patients in the high HICDI group, treatment strategies may need to more actively combine immune modulators or targeted therapeutic drugs to break the immunosuppressive state and enhance treatment outcomes, whereas for patients in the low HICDI group, targeted metabolic therapies can be explored based on their metabolic characteristics, providing new ideas and methods to enhance patient prognosis (63).

To preliminarily evaluate the clinical applicability and generalizability of the HICDI model, we performed external validation across multiple independent treatment cohorts. The results suggest that the model may have certain reference value in predicting treatment response and prognosis. TIDE and IPS analyses indicated that the low HICDI score group might exhibit a better response to immunotherapy, and a higher IPS score was associated with favorable prognosis. Furthermore, additional validation in a transarterial chemoembolization (TACE) treatment cohort suggested that the HICDI model may possess potential prognostic predictive value not only for immunotherapy but also for locoregional interventional therapy. Collectively, HICDI may help identify immune−sensitive populations and enable non−invasive risk stratification, providing preliminary exploratory evidence for the clinical translation of precision oncology.

GSEA analysis revealed that pathways associated with EMT, cell cycle, and proliferation were predominantly activated in the high HICDI group. This finding is highly consistent with the phenotype characterized by a high proliferation rate of tumor cells in the high HICDI group. Furthermore, regarding clinical treatment guidance, drug sensitivity analysis revealed that the high HICDI group exhibited sensitivity to common drugs, including gemcitabine and sorafenib. The elevated highly proliferative traits of tumor cells in the high HICDI group may render patients in this group more likely to benefit from chemotherapeutic drugs. To enhance clinical treatment options for patients in the high HICDI group, we identified drugs, including clofarabine, as potential therapeutic targets by aligning drug mechanisms of action with the molecular characteristics of the high HICDI group, utilizing drug sensitivity data from tumor drug databases GDSC and CTRP (64). Future research will require more clinical trials to confirm the potential roles of these drugs in monotherapy and combination therapy for HCC in the high HICDI group. Furthermore, utilizing single-cell analysis and spatial transcriptomics, we examined the distribution of HICDI genes within the TME. As expected, these genes were primarily situated in highly proliferative cell clusters, which is highly consistent with our previous findings that the high HICDI group correlates with the activation of cell cycle and proliferation pathways. This study validated that disruption of the key gene KIF2C results in reduced proliferation and migration/invasion capabilities of HCC cells through CCK8 and Transwell assays. Additionally, we discovered through RNA sequencing that KIF2C may influence apoptosis in HCC cells through the PI3K/AKT signaling pathway.

Overall, our research findings indicate that HICDI exhibits potential utility in predicting the genomic patterns and immunotherapy response of patients with HCC and even patients with other cancers. This study has several limitations. First, the current results are mainly based on retrospective public cohort data, which may be subject to cross-platform heterogeneity as well as selection and information biases inherent to retrospective cohorts. Additionally, the lack of prospective multicenter clinical trial data to support generalizability across different clinical settings means that further validation is required for clinical translation. Second, the mechanisms underlying poor prognosis in patients with high HICDI risk need to be further explored through wet−lab experiments. Moreover, only single gene validation has been performed for KIF2C, failing to fully reflect the biological functions of the complete signature. Further *in vivo* studies are needed to investigate the functional roles of other risk genes in hepatocellular carcinoma. Finally, the conclusions of this study are primarily based on HCC data, and their application in non−HCC immunotherapy settings requires dedicated validation.

## Data Availability

The original contributions presented in the study are included in the article/**Supplementary Material**. Further inquiries can be directed to the corresponding author.
