# Supplementary figures and images for: Immune - cell death index in hepatocellular carcinoma: a multi-omics and machine learning study for prognosis and immunotherapy prediction
Source: Front Immunol. 2026 Jun 11;17:1776723. doi: 10.3389/fimmu.2026.1776723 (PMC13294073; doi:10.3389/fimmu.2026.1776723)

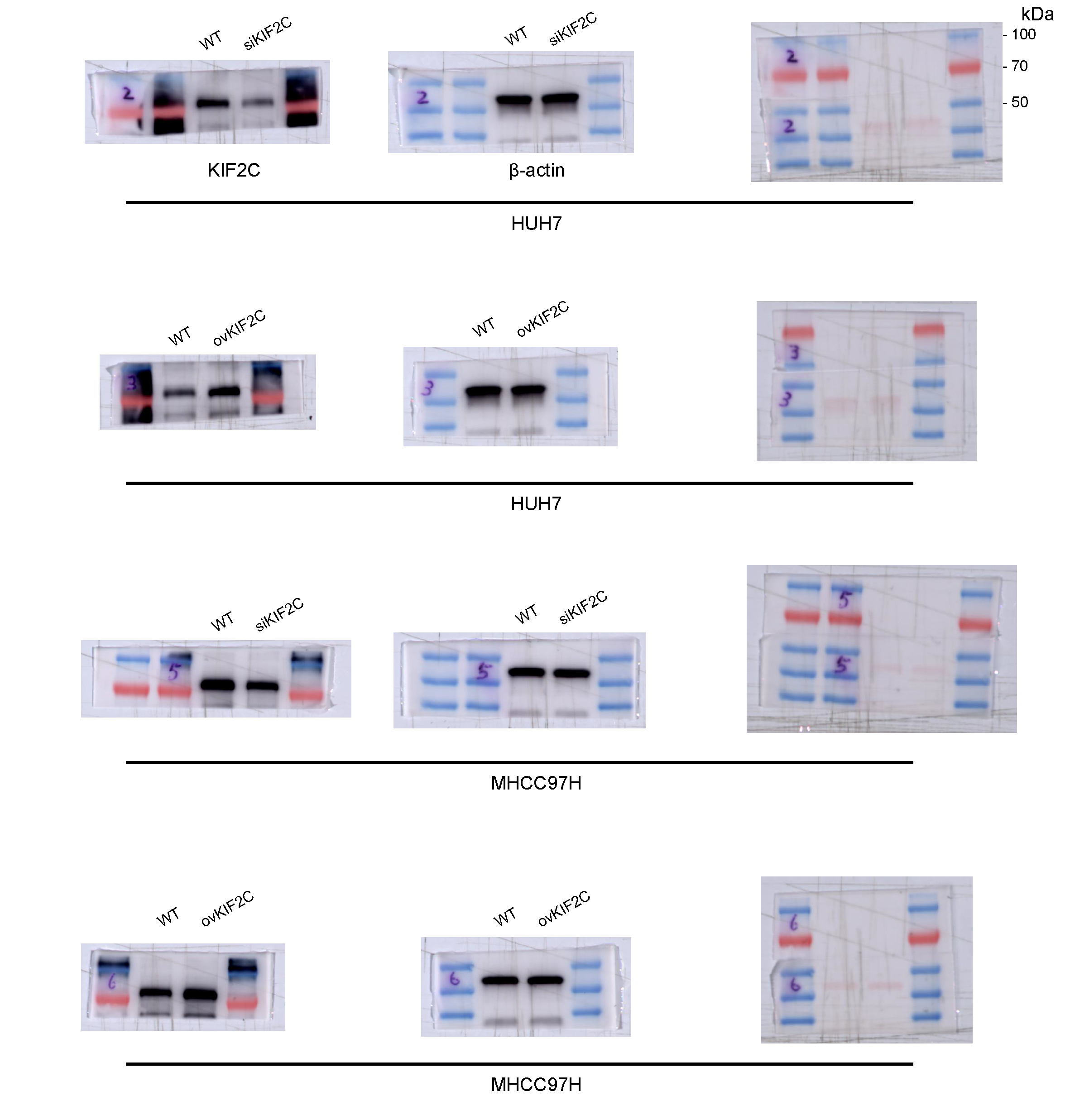

Supplement: Supplementary file 1 [file SupplementaryFile1.tif]
