# Supplementary material for: Immune - cell death index in hepatocellular carcinoma: a multi-omics and machine learning study for prognosis and immunotherapy prediction
Source: Front Immunol. 2026 Jun 11;17:1776723. doi: 10.3389/fimmu.2026.1776723 (PMC13294073; doi:10.3389/fimmu.2026.1776723)
Supplement: Supplementary file 2 [file Table1.docx]

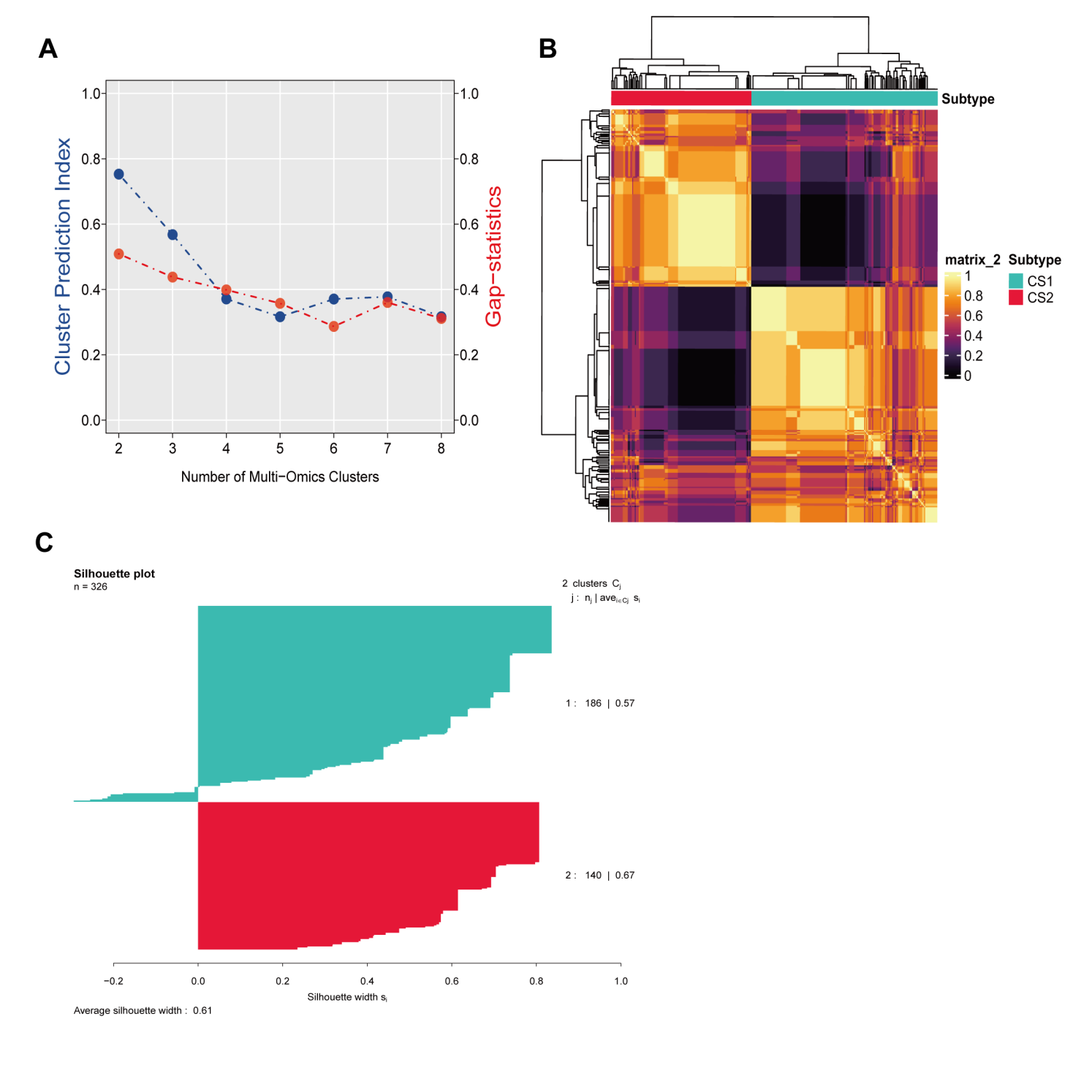


**Supplementary Figure 1**

1. Determination of clustering coefficients for TCGA-LIHC multi-omics data. (B) Consensus heatmap constructed using various integrated multi-omics clustering algorithms. (C) Evaluation of sample homogeneity through silhouette scores derived from consensus ensemble results.

**
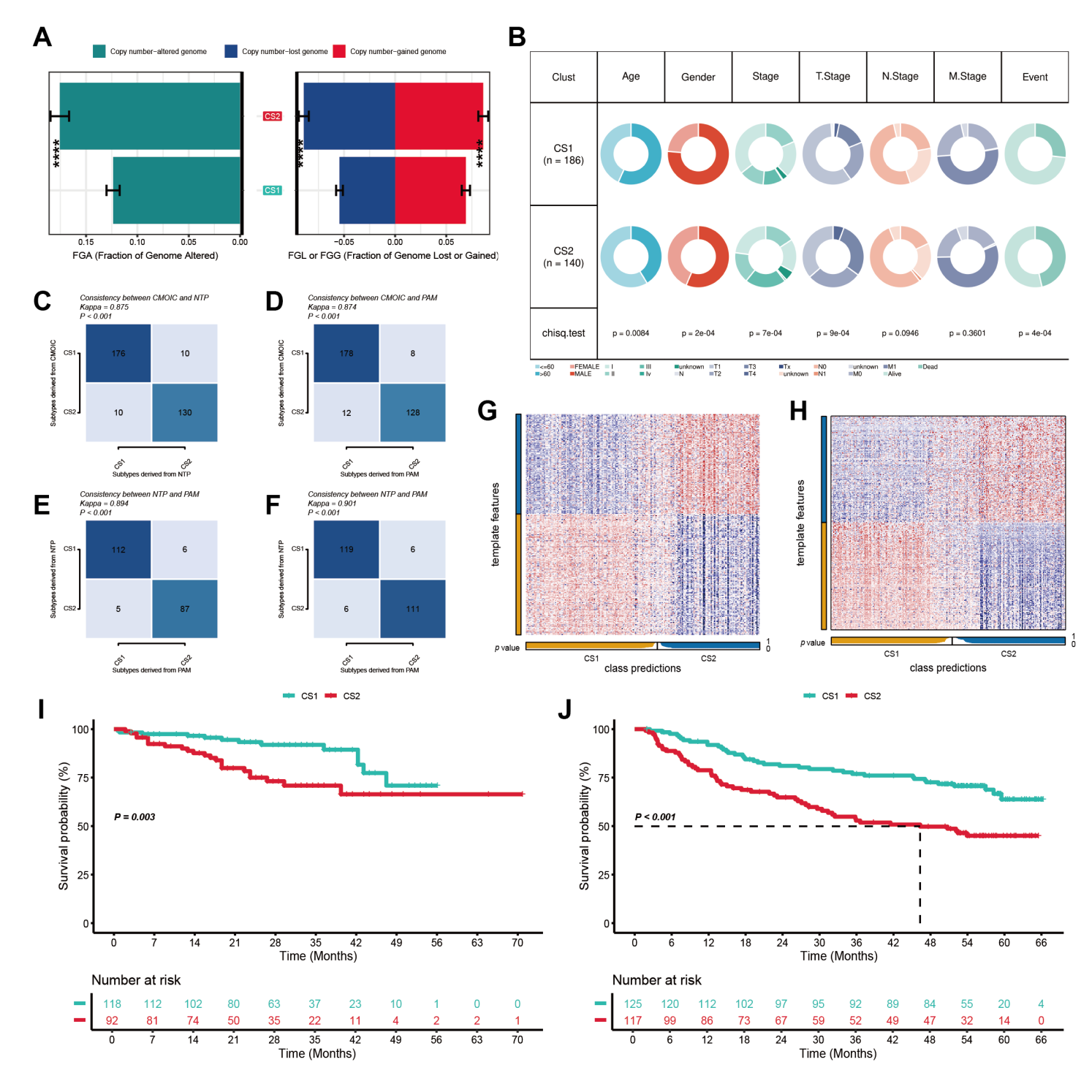
**

**Supplementary Figure 2**

Clinical characteristics and evaluation of subtypes. (A) Comparison of the proportions of genomic alterations, including losses and gains, across different subtypes. (B) Clinical characteristics of different subtypes. (C) Consistency between the identified subtypes and NTP in the TCGA cohort. (D) Consistency between the subtypes and PAM in the TCGA cohort. (E) Consistency between NTP and PAM in the ICGC-LIRI cohort. (F) Consistency between NTP and PAM in the GSE14520 cohort. (G) Validation of HCC subtypes in the ICGC-LIRI cohort using the nearest template approach. (H) Validation of HCC CSs in the GSE14520 cohort using the nearest template approach. (I) Survival analysis of different subtypes in the ICGC-LIRI cohort. (J) Survival analysis of different subtypes in the GSE14520 cohort.


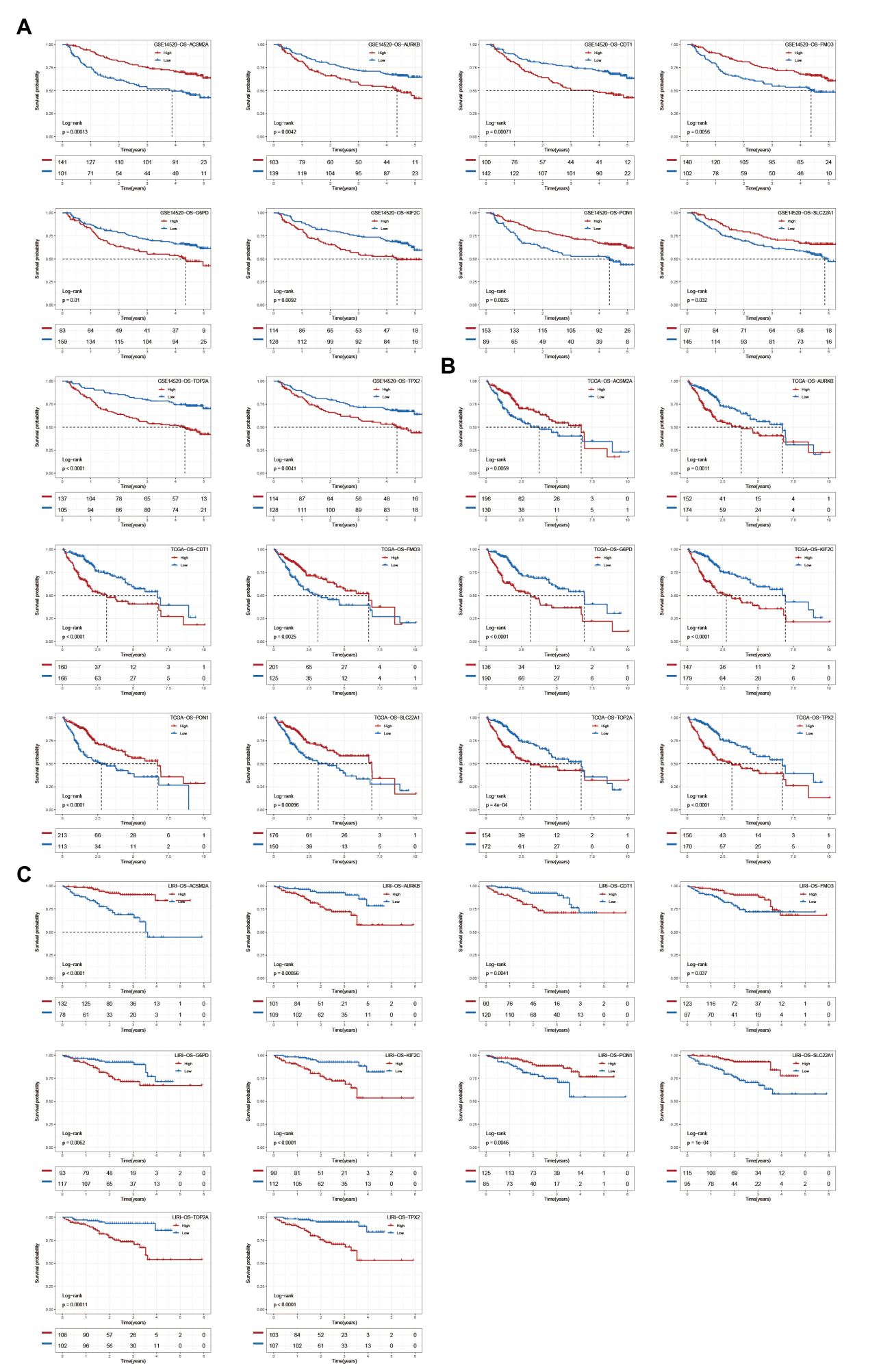


**Supplementary Figure 3**

1. C). Kaplan-Meier analysis of HICDI genes in TCGA, LIRI and GSE14520 Datasets


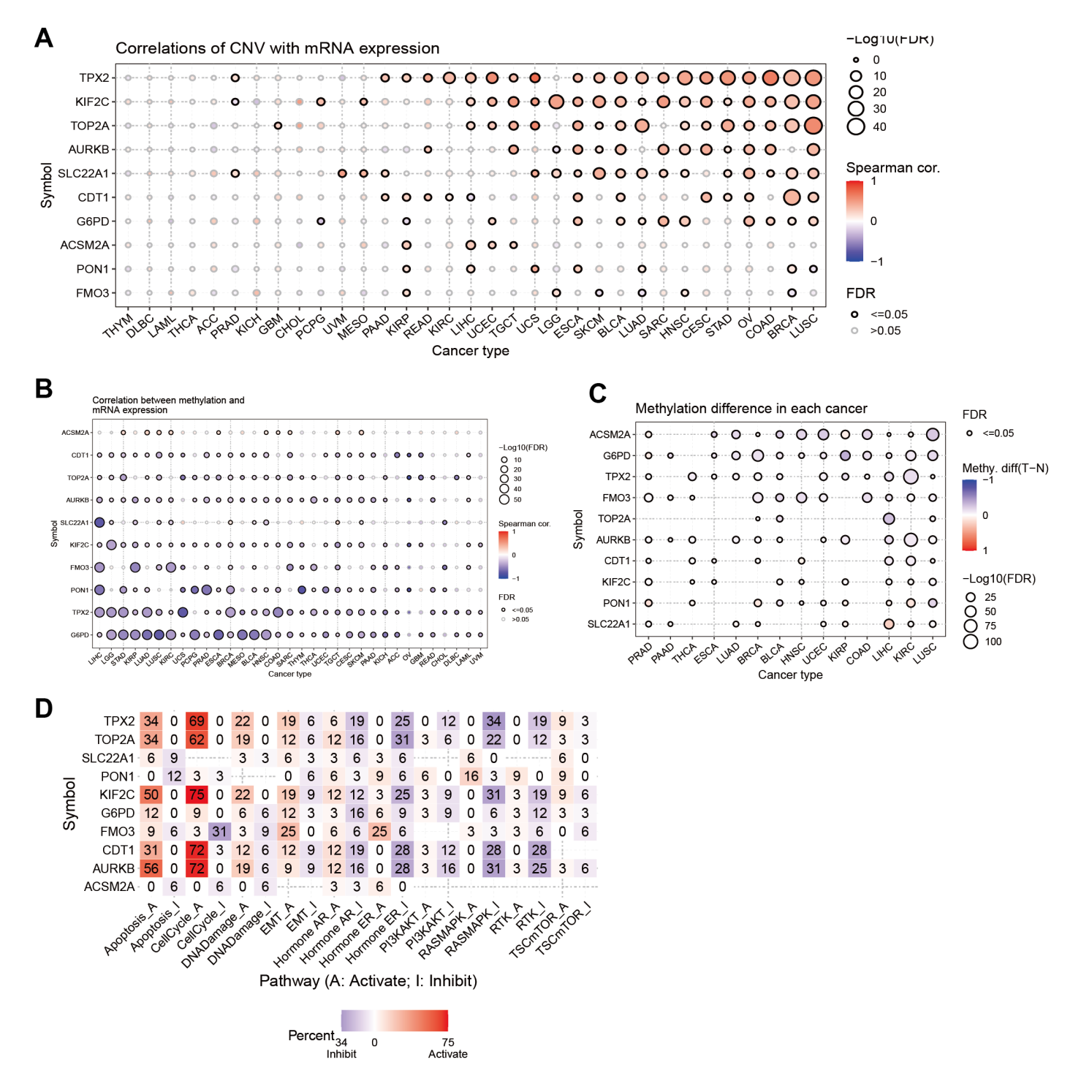


**Supplementary Figure 4**

1. CNV-mRNA Expression Correlations of HICDI Genes Across Diverse Cancer Types. (B) Methylation-mRNA Expression Correlations of HICDI Genes. (C)  Cancer Methylation Differences Among HICDI Genes. (D) Pathway Activation/Inhibition Patterns Mediated by HICDI Genes.


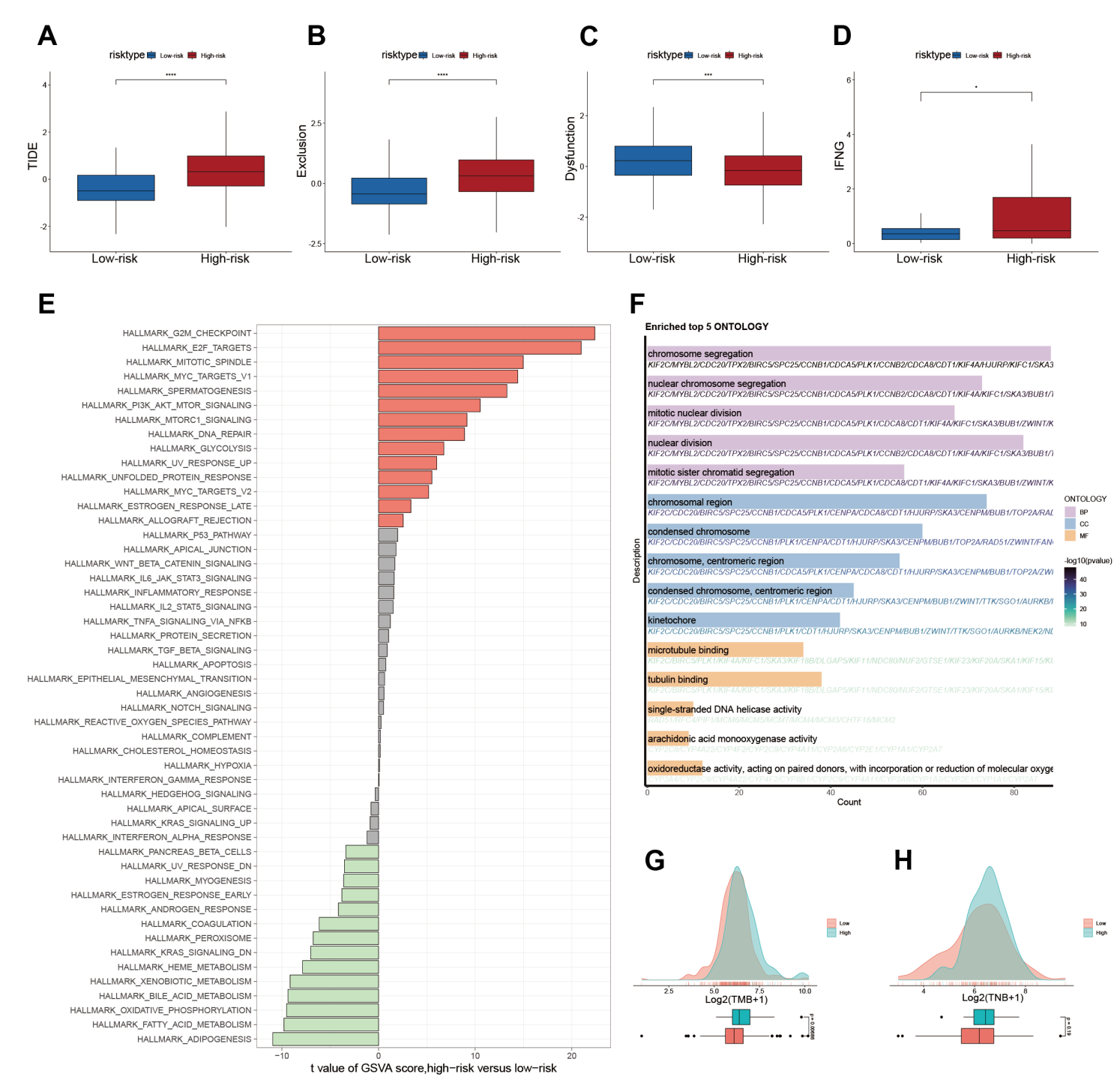


**Supplementary Figure 5**

Pathway enrichment and molecular characteristics. (A–D) Differences in the distribution of immune-related indicators (TIDE, exclusion, dysfunction, and IFNG) between high- and low-risk groups. (E) Results of GSVA analysis for Hallmark pathways. (F) Results of functional enrichment analysis for high- and low-risk groups. (G-H) Distribution of TMB and tumor neoantigen burden (TNB) in patients with high and low-risk scores.


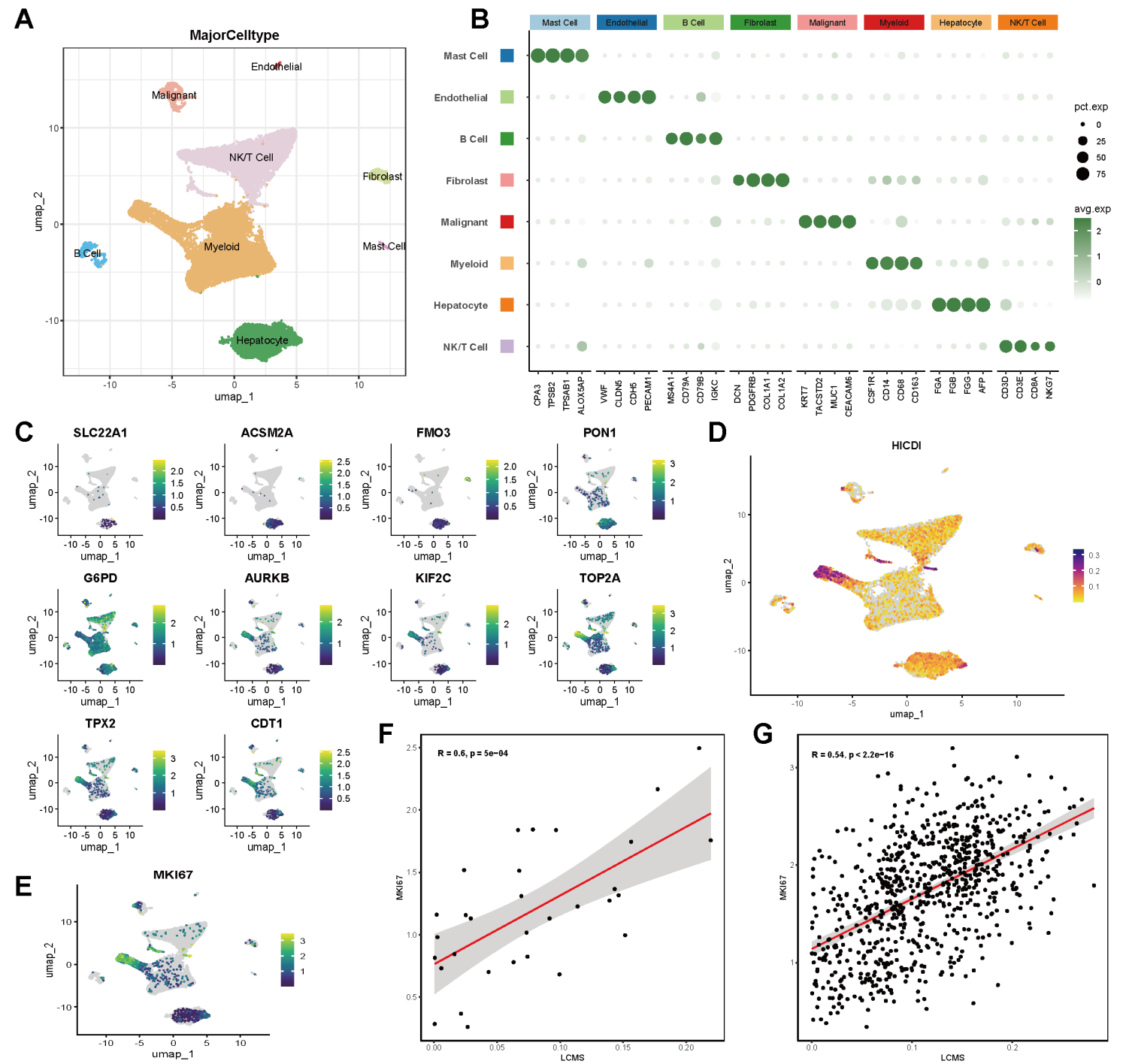


**Supplementary Figure 6**

Single-cell transcriptomics validation of HICDI-related genes (A) Single-cell analysis of GSE166635. (B) Bubble chart display of cellular markers. (C) Distribution of HICDI-associated genes at the single-cell level. (D) Distribution of HICDI scores at the single-cell level. (E) Distribution of the classical cell proliferation marker MKI67 at the single-cell level. (F-G) Correlation between HICDI scores and MKI67 in malignant cells and circulating myeloid cells at the single-cell level.


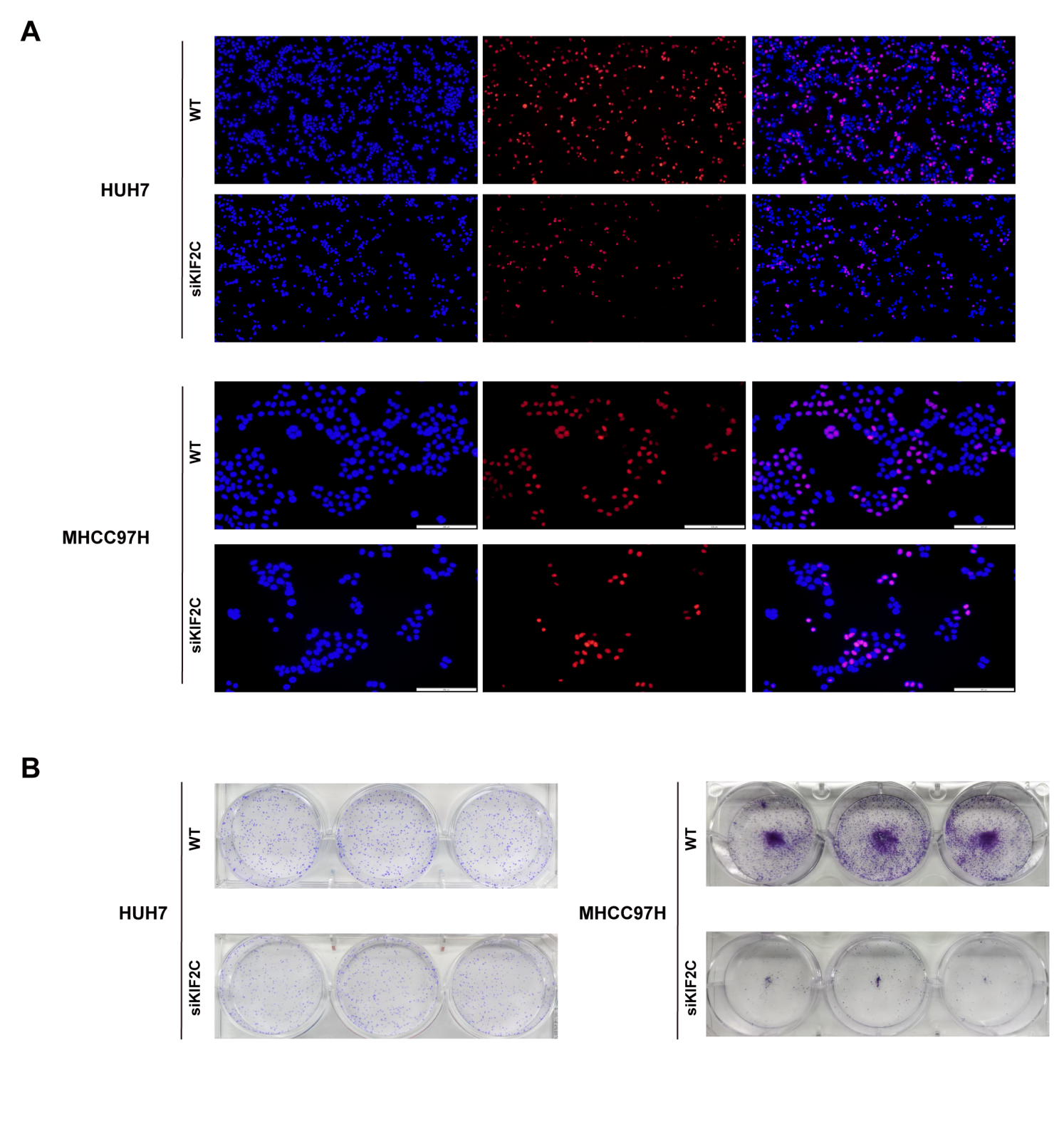
**Supplementary Figure 7**

1. EdU assay for assessing cell proliferation in KIF2C-knockdown HUH7 and MHCC97H cell lines（n=3).
2. Colony formation assay of KIF2C-knockdown HUH7 and MHCC97H cell lines（n=3).
